# Cannabinoid Attenuation of Intestinal Inflammation in Chronic SIV-Infected Rhesus Macaques Involves T Cell Modulation and Differential Expression of Micro-RNAs and Pro-inflammatory Genes

**DOI:** 10.3389/fimmu.2019.00914

**Published:** 2019-04-30

**Authors:** Vinay Kumar, Workineh Torben, Joshua Mansfield, Xavier Alvarez, Curtis Vande Stouwe, Jian Li, Siddappa N. Byrareddy, Peter J. Didier, Bapi Pahar, Patricia E. Molina, Mahesh Mohan

**Affiliations:** ^1^Nektar Therapeutics, South San Francisco, CA, United States; ^2^Department of Biological Sciences, LSU, Alexandria, LA, United States; ^3^Division of Comparative Pathology, Tulane National Primate Research Center, Covington, LA, United States; ^4^Department of Physiology, LSUHSC, New Orleans, LA, United States; ^5^Department of Global Biostatistics and Data Science, Tulane University School of Public Health and Tropical Medicine, New Orleans, LA, United States; ^6^Department of Pharmacology and Experimental Neuroscience, University of Nebraska Medical Center, Omaha, NE, United States; ^7^LSUHSC Alcohol and Drug Abuse Center, New Orleans, LA, United States

**Keywords:** THC, SIV, rhesus macaque, intestinal inflammation, micro-RNA

## Abstract

Cannabis use is frequent in HIV-infected individuals for its appetite stimulation and anti-inflammatory effects. To identify the underlying molecular mechanisms associated with these effects, we simultaneously profiled micro-RNA (miRNA) and mRNA expression in the colon of chronically simian immunodeficiency virus (SIV)-infected rhesus macaques administered either vehicle (VEH/SIV; *n* = 9) or Δ^9^-tetrahydrocannabinol (Δ^9^-THC; THC/SIV; *n* = 8). Pro-inflammatory miR-130a, miR-222, and miR-29b, lipopolysaccharide-responsive miR-146b-5p and SIV-induced miR-190b were significantly upregulated in VEH/SIV rhesus macaques. Compared to VEH/SIV rhesus macaques, 10 miRNAs were significantly upregulated in THC/SIV rhesus macaques, among which miR-204 was confirmed to directly target MMP8, an extracellular matrix-degrading collagenase that was significantly downregulated in THC/SIV rhesus macaques. Moreover, THC/SIV rhesus macaques failed to upregulate pro-inflammatory miR-21, miR-141 and miR-222, and alpha/beta-defensins, suggesting attenuated intestinal inflammation. Further, THC/SIV rhesus macaques showed higher expression of tight junction proteins (occludin, claudin-3), anti-inflammatory *MUC13*, keratin-8 (stress protection), *PROM1* (epithelial proliferation), and anti-HIV *CCL5*. Gomori one-step trichrome staining detected significant collagen deposition (fibrosis) in the paracortex and B cell follicular zones of axillary lymph nodes from all VEH/SIV but not in THC/SIV rhesus macaques, thus demonstrating the ability of Δ^9^-THC to prevent lymph node fibrosis, a serious irreversible consequence of HIV induced chronic inflammation. Furthermore, using flow cytometry, we showed that Δ^9^-THC suppressed intestinal T cell proliferation/activation (Ki67/HLA-DR) and PD-1 expression and increased the percentages of anti-inflammatory CD163^+^ macrophages. Finally, while Δ^9^-THC did not affect the levels of CD4^+^ T cells, it significantly reduced absolute CD8^+^ T cell numbers in peripheral blood at 14 and 150 days post-SIV infection. These translational findings strongly support a role for differential miRNA/gene induction and T cell activation in Δ^9^-THC-mediated suppression of intestinal inflammation in HIV/SIV and potentially other chronic inflammatory diseases of the intestine.

## Introduction

Cannabis use for both medical and recreational purposes is frequently reported in persons living with HIV (PLWH) (1–3). Both natural and synthetic forms of Δ^9^-tetrahydrocannabinol (Δ^9^-THC) have been demonstrated to stimulate appetite, increase body weight, ameliorate adverse effects of combination anti-retroviral therapy (cART), and collectively improve the overall well-being of HIV-infected individuals (1–3). Medical surveys and small-scale clinical studies have revealed frequent cannabis use by patients with inflammatory bowel disease (IBD) and it's potential to alleviate diarrhea, abdominal pain, and anorexia (4, 5). Recently, two studies found significantly lower levels of circulating CD16^+^ monocytes and plasma IP-10 levels (6) and reduced frequencies of human leukocyte antigen (HLA)-DR^+^CD38^+^CD4^+^ and CD8^+^ T cell frequencies (7) in marijuana smoking HIV-infected patients compared to non-marijuana smokers. Nevertheless, detailed scientific knowledge underlying cannabinoid action in alleviating inflammation and immune modulation, particularly in the gastrointestinal (GI) tract, a central organ of cannabinoid signaling (5), and the primary site of HIV/SIV replication (8) are limited.

Cannabinoids mediate their effects by binding to two major G protein-coupled receptors, namely, CB1 expressed predominantly in brain and CB2 on immune cells (4, 5). Most notably, the discovery of CB2 on immune cells in addition to enhancing our understanding of cannabinoid action has identified additional biomedical effects with potential implications in inflammation and immune modulation (4, 5). In this context, activation of CB2 receptors has been demonstrated to alter immune responses in several ways that include but are not limited to induction of apoptosis, inhibition of pro-inflammatory cytokine expression, and shift from Th1 to a Th2 immune response and more recently, the induction of myeloid-derived suppressor and T-regulatory cells (9).

Inflammation and immune dysregulation directly or indirectly lead to most of the morbidity and mortality in HIV infection, even in virally suppressed PLWH (8). One of the proposed mechanisms driving chronic inflammation and systemic immune activation in HIV/SIV infection is intestinal microbial translocation, a pathogenic event triggered by persistent intestinal inflammation (8). In this context, chronic administration of Δ^9^-THC to SIV-infected rhesus macaques alleviated infection-induced inflammation and prolonged survival (10). To understand the mechanisms underlying the anti-inflammatory effects of Δ^9^-THC, we found regulation of micro-RNA (miRNA) expression to be one mechanism by which cannabinoids reduced intestinal inflammation in acutely SIV-infected rhesus macaques (11). Nevertheless, an important unanswered question was whether modulation of epigenomic mechanisms persisted during untreated chronic SIV infection and was paralleled by differential modulation of intestinal gene expression and T cell (activation and PD-1 expression) and macrophage dynamics. Consistent with its immunomodulatory effects (11), long-term Δ^9^-THC administration effectively inhibited classical pro-inflammatory miRNA and gene expression in colons of chronically SIV-infected rhesus macaques. *In vitro* studies showed that miR-204, a miRNA upregulated in the colon of THC/SIV rhesus macaques could potentially target and downregulate the expression of *MMP8*, an extracellular matrix-degrading collagenase produced by both neutrophils and colonic epithelium (CE) (12). Interestingly, the anti-inflammatory effects of cannabinoids extended beyond the intestine as Δ^9^-THC prevented peripheral lymph node fibrosis. Finally, and most importantly, long-term Δ^9^-THC administration suppressed intestinal T cell proliferation/activation and programmed death-1 (PD-1) expression, increased intestinal CD163 anti-inflammatory macrophages, and reduced CD8^+^ T cell expansion in peripheral blood without any adverse effects.

## Materials and Methods

### Animal Care, Ethics, and Experimental Procedures

All experiments using rhesus macaques were approved by the Tulane and LSUHSC Institutional Animal Care and Use Committee (Protocol Nos- 3581 and 3781). The Tulane National Primate Research Center (TNPRC) is an Association for Assessment and Accreditation of Laboratory Animal Care International-accredited facility (AAALAC #000594). The NIH Office of Laboratory Animal Welfare assurance number for the TNPRC is A3071-01. All clinical procedures, including administration of anesthesia and analgesics, were carried out under the direction of a laboratory animal veterinarian. Animals were anesthetized with ketamine hydrochloride for blood collection procedures. Intestinal pinch biopsies were collected by laboratory animal veterinarians. Animals were pre-anesthetized with ketamine hydrochloride, acepromazine, and glycopyrrolate, intubated and maintained on a mixture of isoflurane and oxygen. All possible measures were taken to minimize the discomfort of all the animals used in this study. Tulane University complies with NIH policy on animal welfare, the Animal Welfare Act, and all other applicable federal, state and local laws.

### Animal Model and Experimental Design

Eighteen age and weight-matched male Indian rhesus macaques were randomly distributed into three groups. Group 1 (*n* = 4) received twice daily injections of vehicle (VEH) (1:1:18 of emulphor: alcohol: saline) and were infected intravenously with 100 times the 50% tissue culture infective dose (100TCID_50_) of SIVmac251. Group-2 (*n* = 8) received twice daily injections of Δ^9^-THC for 4 weeks prior to SIV infection. Group 3 (*n* = 6) served as uninfected controls. To obtain adequate statistical power, five SIV-infected animals (FT11, GH25, HB31, GA19, and HD08) that did not receive VEH treatments were added to the VEH/SIV group increasing the group size to nine. However, vehicle treatment alone is unlikely to influence pro-inflammatory signaling in the colon. The lack of effect of vehicle on inflammatory gene expression is clear from the high normalized signal intensity and significantly (*p* < 0.05) low delta CT (ΔC_T_) values for inflammation-induced *defensin-alpha 2* (*DEFA2* or *MNP2*) in the colon of SIV-infected rhesus macaques that did or did not receive vehicle compared to uninfected controls (Figure S1 in Supplementary Material). In contrast, the normalized signal intensity and ΔC_T_ values for *MNP2* in the colon of SIV-infected rhesus macaques that received Δ^9^-THC are not different from uninfected controls. Later, an additional eight age-matched male VEH/SIV (*n* = 4) and THC/SIV (*n* = 4) rhesus macaques were used exclusively for T cell and macrophage immunophenotyping studies, using intestinal pinch biopsies collected longitudinally during the course of the infection (Table 1). Chronic administration of Δ^9^-THC or VEH was initiated 4 weeks before SIV infection at 0.18 mg/kg as used in previous studies (10, 11). This dose of Δ^9^-THC was found to eliminate responding in a complex operant behavioral task in almost all animals (13). The dose was subsequently increased for each subject to 0.32 mg/kg, over a period of ~2 weeks when responding was no longer affected by 0.18 mg/kg on a daily basis (i.e., tolerance developed), and maintained for the duration of the study. The optimization of the Δ^9^-THC dosing in rhesus macaques accounts for the development of tolerance during the initial period of administration. Because in our previously published studies (10, 11) this dose of Δ^9^-THC showed protection, the same dose was used in this study. The 0.32 mg/kg dose was also shown to be effective in SIV-infected rhesus macaques of Chinese origin (14). SIV levels in plasma and intestine were quantified by using the TaqMan One-Step Real-time RT-qPCR assay that targeted the LTR gene (15–18). At necropsy, colon segments were split open and luminal contents were first removed by washing with sterile PBS after which small 1 cm^2^ pieces were collected in RNAlater (Thermo Fisher Scientific, Waltham, MA) for total RNA extraction.

**Table 1:** Animal IDs, SIV inoculum, duration of infection, viral loads and colon histopathology in vehicle or delta-9-tetrahydrocannabinol (Δ^9^-THC) treated chronic SIV-infected and uninfected rhesus macaques.

**Animal ID**	**SIV inoculum**	**Duration of infection**	**Plasma viral loads 10^**6**^/mL**	**Colon viral loads 10^**6**^/mg RNA**	**Colon histopathology**	**Opportunistic infections**
**CHRONIC SIV-INFECTED AND VEHICLE Treated (GROUP 1) FOR MIRNA AND GENE EXPRESSION STUDIES**						
FT11#	SIVmac251	145	500	2,075	Mild colitis	ND
GH25#	SIVmac239	148	30	3,418	Mild suppurative colitis	ND
HB31#	SIVmac251	180	3,000	200	Lymphoid hyperplasia	ND
GA19#	SIVmac251	180	100	600	Lymphoid hyperplasia	ND
HD08#	SIVmac251	90	37	3,000	Moderate colitis/cryptitis	ND
IH96#	SIVmac251	180	0.1	786	Lymphoid hyperplasia	ND
HV48#	SIVmac251	150	4	147	ND	ND
IN24	SIVmac251	180	9.4	29	ND	ND
JC81	SIVmac251	180	0.38	320	ND	ND
**CHRONIC SIV-INFECTED AND VEHICLE TREATED (GROUP 1) FOR IMMUNOPHENOTYPING STUDIES**						
JH47^*^	SIVmac251	180	2	300	ND	ND
JR36^*^	SIVmac251	180	0.5	20	ND	ND
JD66^*^	SIVmac251	180	0.04	4	ND	ND
IV95^*^	SIVmac251	180	0.02	2	ND	ND
**CHRONIC SIV-INFECTED AND Δ^9^-THC TREATED (GROUP 2) FOR MIRNA AND GENE EXPRESSION STUDIES**						
GV60#	SIVmac251	180	18.9	6,726	ND	ND
HT48#	SIVmac251	150	260	9,360	ND	ND
IA83#	SIVmac251	180	1.5	1,261	ND	ND
IH69#	SIVmac251	180	0.06	93.8	ND	ND
HI09	SIVmac251	180	0.01	3.0	ND	ND
JB82	SIVmac251	180	7.7	970	ND	ND
IA04	SIVmac251	150	0.66	35	Mild colitis	ND
AL6094#	SIVmac251	300	NA	40	ND	ND
**CHRONIC SIV-INFECTED AND Δ^9^-THC TREATED (GROUP 2) FOR IMMUNOPHENOTYPING STUDIES**						
JI45^*^	SIVmac251	180	3	10	ND	ND
JT80^*^	SIVmac251	180	1	300	ND	ND
JC85^*^	SIVmac251	180	0.02	1	ND	ND
IV90^*^	SIVmac251	180	0.02	10	ND	ND
**UNINFECTED CONTROLS (GROUP 3)**						
EL66	NA	NA	NA	NA	NA	NA
EH70	NA	NA	NA	NA	NA	NA
EH80	NA	NA	NA	NA	NA	NA
HT22	NA	NA	NA	NA	NA	NA
HF54	NA	NA	NA	NA	NA	NA
HR42	NA	NA	NA	NA	NA	NA

NA, Not applicable; ND, None detected.

* Denotes animals used for immunophenotyping studies, # Denotes animals used for gene (mRNA profiling) expression studies.

All animals including uninfected controls with exception of the eight denoted by an asterisk (^*^) were used for microRNA profiling.

*Note that all five vehicle untreated (FT11-HD08) and only one out of eight vehicle treated (IH96-IV95) macaques showed colon histopathology. However, normalized microarray signal intensity and delta C_T_ values for MNP2, also known as defensin alpha-2 (DEFA2) in Figure S1 showed significant upregulation of MNP2 in vehicle treated (IH96, HV48, IA24, JC81) and untreated (FT11, GH25, HB31, GA19, HD08) macaques suggesting vehicle treatment does not influence inflammatory gene expression in colon despite the absence of detectable histopathological lesions*.

### Global micro-RNA and Gene Expression Profiling

Micro-RNA and mRNA expression profiling were performed using TaqMan OpenArray Human Micro-RNA panels (Thermo Fisher) (11, 18) and whole rhesus macaque 4 × 44 K Gene Expression Microarray V2 (Agilent Technologies), respectively. Briefly, total RNA from colon tissue was isolated using the miRNeasy total RNA isolation kit (Qiagen Inc, CA) following the manufacturer's protocol. Approximately 100 ng total RNA was first reverse transcribed using the micro-RNA reverse transcription reaction kit (Thermo Fisher).

Briefly, two master mixes representing either OpenArray panel (panel A and panel B) were prepared for each RNA sample, which consisted of the following reaction components: 0.75 μL MegaPlex RT primers (10x), 0.15 μL dNTPs with dTTP (100 mM), 1.50 μL MultiScribe™ Reverse Transcriptase (50 U/μL), 0.75 μL 10x RT Buffer, 0.90 μL MgCl_2_ (25 mM), 0.09 μL RNase Inhibitor, 0.35 μL nuclease-free water (20 U/μL). Three microliters of total RNA (100 ng) were loaded into appropriate wells of a 96-well plate together with 4.5 μL of the RT reaction master mix. After a brief spin and 5 min of incubation on ice, samples in the 96-well plate were subjected to the following thermal cycling conditions on the ABI 7900 HT Fast PCR system: standard or max ramp speed, 16°C for 2 min, 42°C for 1 min, 50°C 1 s (40 cycles), 85°C 5 min (hold), 23°C (hold). Immediately after thermal cycling, the 96-well plate containing cDNA was stored at −80°C.

For pre-amplification, 2.5 μL of the cDNA from each sample was mixed with a total of 22.5 μL of pre-amplification reaction master mix consisting of 12.5 μL TaqMan® PreAmp Master Mix (2x), 2.5 μL Megaplex™ PreAmp Primers (10x) and 7.5 μL nuclease-free water in a 96-well plate. After a brief vortex and spin, samples were subjected to the following thermal cycling conditions on the ABI 7900 HT Fast PCR system: standard or max ramp speed, hold 95°C 10 min; hold 55°C 2 min; hold 72°C 2 min; 12 cycles at 95°C 15 s, and 60°C 4 min; hold 4°C. The pre-amplified product was diluted 40 times by mixing 4 μL of the pre-amplified product with 156 μL of 0.1x TE pH 8.0 and loaded onto TaqMan® OpenArray® human micro-RNA plates for processing using the QuanStudio™ 12K Flex Real-Time PCR system (Thermo Fisher).

Transcriptome profiling and data analysis were performed by Arraystar Inc (Baltimore, USA). Acquired array images were analyzed using Rhesus Agilent Feature Extraction software (version 11.0.1.1). Quantile normalization and subsequent data processing were performed using the GeneSpring GX v12.1 software (Agilent Technologies). After quantile normalization of the raw data, genes that did not have flags were chosen for further data analysis. Microarray analysis was performed using four to seven individual animals as biological replicates per group: VEH/SIV group (FT11, GH25, GA19, HB31, HD08-90D, IH96, HV48), THC/SIV group (A2L0694, GV60, HT48, IA83, IH69), and uninfected control group (EL66, EH70, EH80, HT22). The differentially expressed genes between two groups were calculated for fold change (|FC| ≥ 1.5) and statistical significance by unpaired *t*-test (*p* ≤ 0.05) and graphically represented on volcano plots.

### Quantitative Real-Time TaqMan and SYBR Green RT-qPCR Assay for OpenArray® Validation

Expression of miR-204 was quantified in CE using the TaqMan micro-RNA predesigned and pre-optimized assays (Thermo Scientific) (15–18). Approximately 200 ng of total RNA was reverse transcribed using the stem-loop primers provided in the predesigned kit in a total volume of 20 μL. Approximately four μL of cDNA was then subjected to 40 cycles of PCR on the ABI 7900 HT Fast PCR System (Thermo Fisher) using the following thermal cycling conditions: 95°C for 10 min followed by 40 repetitive cycles of 95°C for 15 s and 60°C for 1 min. As a normalization control for RNA loading, parallel reactions in triplicate wells to amplify RNU48 were run in the same multi-well plate. Comparative real-time PCR was performed in duplicate wells including no-template controls and relative change in gene expression was calculated using the comparative CT method. mRNA expression of occludin (*OCLN*), claudin-3 (*CLDN3*), and peroxisome proliferator activator receptor gamma (*PPAR*γ) in jejunal epithelium and defensin alpha 2 (*MNP2* or *DEFA2*) in whole colon tissue was quantified by RT-qPCR using the Power SYBR Green RNA to C_T_ kit (Thermo Fisher). Each RT-qPCR reaction (20 μl) contained the following: 2X Power SYBR Green Master Mix without uracil-N-glycosylase (12.5 μl), forward and reverse primer [*PPAR*γ (For-5′-ATCGCCCAGGTTTGCTGAAT-3′, Rev-5′-GACTCAGGGTGGTTCAGCTTC-3′) and *OCLN* (For-5′-CATTGCCATCTTTGCATGTGT-3′, Rev-5′- GGTAGCCTACACTACCTCCTATAA-3′), *CLDN3* (For-5′- TCATCGGCAGCAACATCATCA-3′, Rev-5′- CGTACACCTTGCACTGCATCT-3′), *MNP2* (For-5′- TCGCTGAGCTTCCTAGATAGA-3′, Rev-5′-CAAGGTACACAGAGCGAAGT-3′), *GAPDH* (For-5′-CAAGAGAGGCATTCTCACCCTGAA-3′, Rev-5′-TGGTGCCAGATCTTCTCCATGTC-3′), and *Beta-actin* (For-5′-CAACAGCCTCAAGATCGTCAGCAA-3′, Rev-5′-GAGTCCTTCCACGATACCAAAGTTGTC-3′) (200 nM) and ~200 ng of total RNA (4 μl). The PCR amplification was carried out in the ABI 7900 HT sequence detection System (Thermo Fisher) using the default thermal cycling conditions for SYBR Green assays. As a normalization control for RNA loading, parallel reactions in the same multi-well plate were performed using GAPDH and β-Actin. Relative changes in gene expression were calculated using the ΔΔC_T_ method. PCR efficiency analysis was performed using serial 10-fold RNA dilution (500, 50, 5, and 0.5 ng of total RNA). The amplification curves for all assays were linear and based on slope values (−3.15 to −3.24) all assays had 103–107% efficiency.

### Colonic Epithelial Cell Isolation

For quantification of miR-204, OCLN, CLDN3, and PPARγ in intestinal (colon and jejunum) epithelial cells, we first separated the epithelial cells from the underlying lamina propria leukocytes (LPLs) and fibrovascular stroma as described previously (15–18). Finally, the intraepithelial lymphocytes (IELs) were separated from the epithelial cells. Briefly, intestinal segments (6–8 cm long) were first incubated with vigorous shaking in Ca^++^Mg^++^ free-HBSS containing 1 mM EDTA for two 30-min incubations at 37°C to separate the intestinal epithelial cells. Two incubations will remove epithelial cells more efficiently than one. The first incubation was done for 30 min with HBSS containing EDTA, the supernatants were removed, and the intestinal pieces were incubated a second time with fresh EDTA containing HBSS. The first incubation will remove around 75–80% of the cells and the remaining 20–25% will detach during the second incubation with fresh media. Following incubation, the epithelial cells in the supernatant were harvested by centrifugation at 500 g for 10 min followed by subjecting the cells to percoll density gradient (35 and 60%) centrifugation to separate IELs. The purified epithelial components were then lyzed in Qiazol (Qiagen Inc, CA) for total RNA extraction.

### Immunoprecipitation and Western Blotting

HEK293 cells were used for luciferase reporter and miR-204 overexpression studies, as these cells abundantly express MMP8 and >95–98% of these cells can be successfully transfected with exogenous nucleic acids using Lipojet® transfection reagent (Signagen Labs, MD). At 96 h post transfection, total protein from transfected (cel-miR-67-3p or miR-204) HEK293 cells was extracted using a lysis buffer (Cell Signaling Technology, Inc, Beverly, MA) containing 20 mM Tris-HCl (pH 7.5), 150 mM NaCl, 1 mM Na_2_EDTA, 1 mM EGTA, 1% Triton, 2.5 mM sodium pyrophosphate, 1 mM beta-glycerophosphate, 1 mM Na_3_VO_4_, 1 μg/ml leupeptin, protease inhibitor cocktail, and phosphatase inhibitor cocktail (Sigma Chemical Company, MO). Total protein was quantified using the Pierce BCA protein assay kit (Thermo Fisher). Approximately, 1,500 μg of total protein extract was immunoprecipitated with ~5 μl of a goat polyclonal antibody against MMP8 (Santa Cruz Biotechnologies, CA), overnight at 4°C followed by incubation with 30 μl (50% w/v) of protein G agarose beads (Invitrogen Corp, CA) at 4°C for 4–5 h. The supernatant was removed and transferred to a new 1.5 mL microcentrifuge tube and immunoprecipitated using goat polyclonal antibody (~5 μl) (Santa Cruz Biotechnologies, CA) to β-actin at 4°C overnight on a shaker. Immunoprecipitated MMP8 and β-actin proteins were heat denatured for 5 min at 100°C in sample loading buffer containing 62.5 mM Tris-HCl, 5% 2-mercaptoethanol, 10% glycerol, 2% SDS, and bromophenol blue, resolved on 8–12% SDS-PAGE gels and transferred to 0.2 μm PVDF membranes (Biorad Laboratories, CA). The membranes were probed with a rabbit polyclonal primary antibody against MMP8 (Abcam), and β-actin (Santa Cruz) followed by the appropriate horseradish peroxidase (HRP) conjugated secondary antibody (Santa Cruz). Membranes were treated with West-Dura chemiluminescent substrate (Pierce Biotechnology Inc, Rockford, IL) for 5 min and the signal was captured using the FluorChem-R imaging system (ProteinSimple) and quantified using ImageJ software (NIH).

### Cloning of 3′-UTR of MMP8 mRNA and Dual-Glo Luciferase Reporter Gene Assay

The 3′ UTR of the rhesus macaque MMP8 mRNA contains a single predicted miR-204 binding site (TargetScan 7.2) (19). Accordingly, a short 34 nucleotide sequence representing the 3′ UTR containing the predicted miR-204 site (5′-GCUGUAUCCAUUUUCAGGAUGUGGUND**AAGGGAA**GUU-3′) was synthesized (IDTDNA Technologies Inc., IA) for cloning into the pmirGLO Dual-Luciferase vector (Promega Corp, Madison, WI) (11, 15–18). A second oligonucleotide with the miRNA binding site deleted (*n* = 7 nucleotides) (5′-GCUGUAUCCAUUUUCAGGAUGUGGGUU-3′) was also synthesized to serve as a negative control. The oligonucleotide sequence was synthesized with a *Pme1* site on the 5′ and *Xba1* site on the 3′ end for directional cloning. The pmirGLO vector was first cut with *Pme1* and *Xba1* restriction enzymes, gel purified and ligated with either wild-type sequence containing the miR-204 binding site (MMP8-wtUTR) or deleted sequence (MMP8-delUTR). HEK293 cells were plated at a density of 2 × 10^4^ cells per well of a 96 well plate. At 60–70% confluence cells were co-transfected with ~100 ng MMP8-wtUTR or MMP8-delUTR luciferase reporter vector and 30 nM miR-204 or negative control (cel-miR-67-3p) mimic using the Lipojet® transfection reagent (Signagen). After 72 h, the Dual Glo luciferase assay was performed according to the manufacturer's recommended protocol (Promega Corp) using the BioTek H4 Synergy plate reader (BioTek, Winooski, VT). The normalized *firefly* to *renilla* ratio was calculated to determine the relative reporter activity. Experiments were performed in 6 replicates and repeated thrice.

### ELISA for Quantifying Plasma Lipopolysaccharide Binding Protein (LBP) Levels

Plasma LBP levels were quantified in duplicate (11) using a commercially available ELISA assay (Biometec, Greifswald, Germany) according to the manufacturer's recommended protocol.

### Quantitation of Mucosal Viral Loads

Total RNA samples from all SIV-infected animals were subjected to a quantitative real-time TaqMan One-step RT-qPCR analysis to determine the viral load in plasma and colon tissue. Briefly, primers and probes specific to the SIV LTR sequence were designed and used in the real-time TaqMan PCR assay. Probes were conjugated with a fluorescent reporter dye (FAM) at the 5′ end and a quencher dye at the 3′ end. Fluorescence signal was detected with an ABI Prism 7900 HT sequence detector (Thermo Fisher). Data were captured and analyzed with Sequence Detector Software Thermo Fisher). Viral copy number was determined by plotting *C*_T_ values obtained from the colon and jejunum samples against a standard curve (y = −3.33x + 40.3) (*r*^2^ = 0.999) generated with *in vitro* transcribed RNA representing known viral copy numbers.

### Flow Cytometry to Quantify Intestinal T Cell Dynamics

Duodenum LPLs and peripheral blood mononuclear cells (PBMCs) were isolated and adjusted to a concentration of 10^7^/ml. For T cell immunophenotyping, ~100 μl aliquots were first stained with live/dead stain (Invitrogen), washed with wash buffer (PBS containing 1% BSA and 7 mM sodium azide), and then surface stained with appropriately diluted, directly conjugated anti-CD3 (SP34-2, BD Biosciences, San Jose, CA), anti-CD4 (L200, BD Biosciences), anti-CD8 (3B5, Invitrogen), Anti-CD14 (M5E2, BD Biosciences), anti-CD45 (D058-1283, BD Biosciences), anti-CD163 (GHI/61, BD Biosciences), anti-HLA-DR (L243, Biolegend, CA), and anti-PD-1 (Programmed Death 1) (EH12.2H7, Biolegend) monoclonal (mAb) antibodies and incubated for 30 min at room temperature (RT) in the dark (20). For Ki67 staining, cells were permeabilized with permeabilization buffer (BD Biosciences) and stained with anti-Ki67 (B56, BD Biosciences) by incubating for 30 min at RT in the dark (21). Samples were finally washed in washed buffer and fixed using BD Stabilizing and fixative buffer (BD Biosciences) and stored in the dark at 4°C overnight for acquisition the next day. Samples were acquired on LSR II flow cytometry equipment (BD Biosciences) and analyzed with FlowJo (Treestar Inc, Ashland, OR). The cells were first gated on singlets followed by CD45, live cells, lymphocytes, CD3^+^ T cells, and then on CD3^+^CD4^+^CD8^+/−^ and CD3^+^CD4^+/−^CD8^+^ T cell subsets (Figure S2 in Supplementary Material) (21, 22). The CD3^−^CD14^−^ cells were further gated to quantify the CD163^+^ population (23). All flow cytometric acquisition was performed by Tulane Flow Cytometry Core Laboratory.

### Quantitative Image Analysis of Lymph Node Sections for Collagen Deposition

Z-fixed paraffin-embedded lymph node sections were stained by Gomori one-step trichrome staining method (24). The images were captured with a CRi multispectral camera (Perkin Elmer) and Nuance software. Ten images were collected from each animal lymph node at 20x magnification. The images were analyzed using Volocity software (v6.3.1) by selecting a circular region of interest (ROI) within the lymphoid follicle and measuring the intensity of blue color. A similar sized ROI was collected from nearby regions as background blue intensity, which was then subtracted from the ROI within the follicle. The values are expressed as Blue intensity per mm^2^.

### Data Analysis and Data Availability

QuantStudio™ run files from all groups were first imported into ExpressionSuite software v1.0.2 (Thermo Fisher) and simultaneously analyzed using the uninfected control group as the calibrator to obtain relative gene expression values. The results from the ExpressionSuite software analysis containing five columns (well, sample, detector, task, and C_T_ values) were saved as a tab-delimited text file which was later imported and analyzed using the Omics Office StatMiner qPCR analysis software, TIBCO Spotfire (Perkin Elmer, Waltham, MA). Omics Office StatMiner Software utilizes the comparative CT (ΔΔCτ) method to rapidly and accurately quantify relative gene expression across many genes and samples. MiRNA expression data were normalized using the global normalization method and analyzed using the non-parametric Wilcoxon's rank sum test. In all experiments, the CT upper limit was set to 28. A *p*-value of less than or equal to 0.05 (≤0.05) was considered significant. Heatmaps were generated using matrix2png software (25). OpenArray TaqMan miRNA (Accession number- GSE121440, https://www.ncbi.nlm.nih.gov/geo/query/acc.cgi?acc=GSE121440) and Agilent gene expression (Accession number- GSE121439, https://www.ncbi.nlm.nih.gov/geo/query/acc.cgi?acc=GSE121439) data have been deposited with GEO.

For individual miRNA RT-qPCR confirmation studies, the VEH/SIV macaque with the highest ΔC_T_ value served as the calibrator/reference and was assigned a value of 1. All differentially expressed miRNAs in the THC/SIV and VEH/SIV groups are shown as an n-fold difference relative to this macaque. This approach was taken mainly to facilitate graphing the control (VEH/SIV) samples so that the variation within this group could be displayed. miRNA RT-qPCR data were analyzed using non-parametric Wilcoxon's rank sum test for independent samples using Omics Office StatMiner qPCR analysis software, TIBCO Spotfire (Perkin Elmer). Lipopolysaccharide binding protein data were analyzed using the Mann-Whitney *U*-test employing the Prism v5 software (GraphPad software). *Firefly/Renilla* ratios were statistically analyzed using one-way ANOVA and *post hoc* testing using the Benjamini-Hochberg method (*p* ≤ 0.05, FDR = 5%). Flow cytometry data were analyzed using linear mixed models with immune-marker outcomes being dependent variables, and treatment status (VEH vs. Δ^9^-THC) and days since the start of treatment (0, 14, 120/150, 180) being independent variables with fixed effects. A random effect for all rhesus macaques was also included. Since multiple animals were included in each specific setup, these animals were considered as repeated measures and their individual effects on the immune-marker outcomes were considered as random components. To eliminate any potential group differences before treatment, baseline values for the immune-marker outcomes were adjusted. Briefly, for each macaque, its baseline value was subtracted from its immune-marker outcomes after the start of treatments, and the resulting differences were used in subsequent analyses. Interactions between status and day were also evaluated. Differences between two post-infection time points were analyzed using Mann-Whitney *U*-test employing the Prism v5 software (GraphPad software). Lymph node image analysis data were graphed using Prism software (v5) and analyzed using non-parametric Mann-Whitney *U*-test.

## Results

### Plasma and Intestinal Viral Loads, CD4^+^ and CD8^+^ T Cell Status and Intestinal Histopathology

All VEH/SIV and THC/SIV rhesus macaques had substantial plasma and colonic viral loads at ~180 days post infection (Table 1). No difference is plasma and colonic viral loads were detected between VEH/SIV and THC/SIV rhesus macaques. Intestinal CD4^+^ T cell data available from three VEH/SIV and six THC/SIV rhesus macaques at necropsy showed marked depletion of this population that was accompanied by a concomitant increase in CD8^+^ T cell percentages (Table S1 in Supplementary Material compared to **Figures 7C,D** at day 0). Longitudinal viral loads, CD4^+^ and CD8^+^ T cell dynamics from a separate cohort are shown further below. Histopathological analysis revealed the presence of severe lymphoid hyperplasia and mild to moderate colitis in 6/13 VEH/SIV and 1/12 THC/SIV rhesus macaques (Table 1). Five VEH/SIV (FT11, GH25, HB31, GA19, HD08, HV48) and two THC/SIV (HT48, IA04) rhesus macaques progressed to AIDS. Consistent with the presence of colonic lesions, the six macaques in the VEH/SIV group (FT11, GH25, HB31, GA19, HD08, and IH96) had higher plasma and colonic viral loads compared to three macaques (HV48, IN24, JC81) that did not have detectable histopathological lesions.

### Δ^9^-THC Administration Significantly Downregulated Pro-inflammatory miRNA Expression in Colon of Chronically SIV-infected Rhesus Macaques

**Figure 1:**
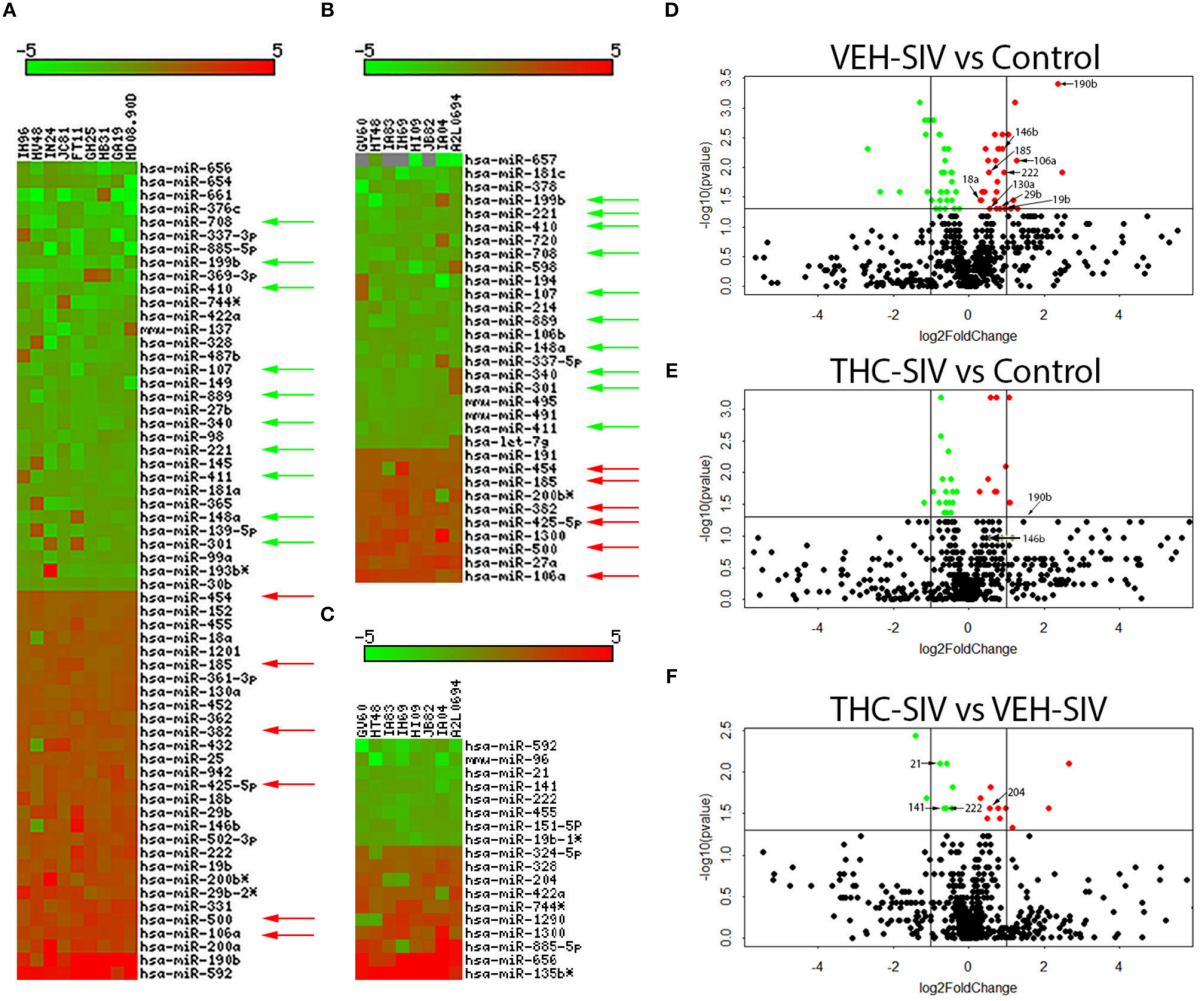
Changes in miRNA expression in whole colon tissue of chronically SIV-infected rhesus macaques administered vehicle (VEH) or delta-9-tetrahydrocannabinol (Δ^9^-THC). The heat map shows all differentially expressed (*p* ≤ 0.05) miRNAs in whole colon tissue of VEH/SIV vs. controls **(A)**, THC/SIV vs. controls **(B)**, and VEH/SIV vs. THC/SIV **(C)**. MiRNA species originating from the opposite arm of the precursor are denoted with an asterisk (^*^). Volcano plot shows the relationship between fold-change (X-axis) and statistical significance (Y-axis) of differentially expressed miRNAs in VEH/SIV **(D)** and THC/SIV **(E)** rhesus macaques relative to controls and in THC/SIV relative to VEH/SIV rhesus macaques **(F)**. The vertical lines in **(D–F)** correspond to 2.0-fold up and down, respectively, and the horizontal line represents *p* ≤ 0.05. The negative log of statistical significance (*p-*value) (base 10) is plotted on the Y-axis, and the log of the fold change base (base 2) is plotted on the X-axis in **(D–F)**. Green and red arrows indicate, respectively, downregulated and upregulated miRNAs common to VEH/SIV and THC/SIV rhesus macaques. The location of miRNAs of interest is denoted with arrows in **(D–F)** without the prefix (miR) due to space limitations.

To determine the epigenomic effects of prolonged Δ^9^-THC administration, we profiled miRNA expression in colons of chronically SIV-infected rhesus macaques receiving either vehicle or Δ^9^-THC. Compared to controls (*n* = 6), 61 (29-up and 32-down) miRNAs were differentially expressed in VEH/SIV rhesus macaques (*n* = 9) (Figure 1A). Tables S2–S4 in Supplementary Material show raw C_T_, fold change and *P*-values for all differentially expressed miRNAs in the colon of VEH/SIV and THC/SIV macaques relative to controls and THC/SIV macaques compared to VEH/SIV macaques, respectively. These included miR-106a, miR-18a, miR-185, miR-222, and miR-29b previously described by us and others to be upregulated in HIV/SIV and other chronic intestinal inflammatory diseases (26–30) (Figure 1D). Notably, expression of LPS-responsive miR-146b-5p (17, 31), peroxisome proliferator-activated receptor gamma (PPARγ) targeting miR-130a (18), and SIV replication induced miR-190b (5.2-fold up) (15, 17) were markedly elevated in VEH/SIV rhesus macaques (Figure 1D). In striking contrast, 50% fewer miRNAs (*n* = 32; 10 up and 22 down) were differentially expressed in colons of THC/SIV rhesus macaques (*n* = 8) compared to controls (*n* = 6) (Figures 1B,E). Approximately 10 miRNAs were commonly down (green arrows) and 6 were upregulated (red arrows), in both VEH/SIV and THC/SIV relative to control uninfected rhesus macaques (Figures 1A,B). Although miR-190b expression was elevated by ~2.7-fold in colons of THC/SIV rhesus macaques, it failed to reach statistical significance (Figure 1E). Another notable difference was the complete absence of miR-146b upregulation (LPS-responsive) in colons of THC/SIV rhesus macaques (Figure 1E). Finally, when comparing VEH/SIV to THC/SIV rhesus macaques, 18 miRNAs (10-up and 8-downregulated) were differentially expressed (Figures 1C,F). Noticeably, the classical pro-inflammatory miR-21 (32), miR-141 (33), and miR-222 (27) previously shown to be markedly upregulated in colons of IBD patients showed significant downregulation (*p* ≤ 0.05) in THC/SIV rhesus macaques.

### Genes Encoding Interferon Stimulated and Intestinal Anti-microbial Defensins Are Significantly Upregulated in VEH/SIV Compared to Control and THC/SIV Rhesus Macaques

**Figure 2:**
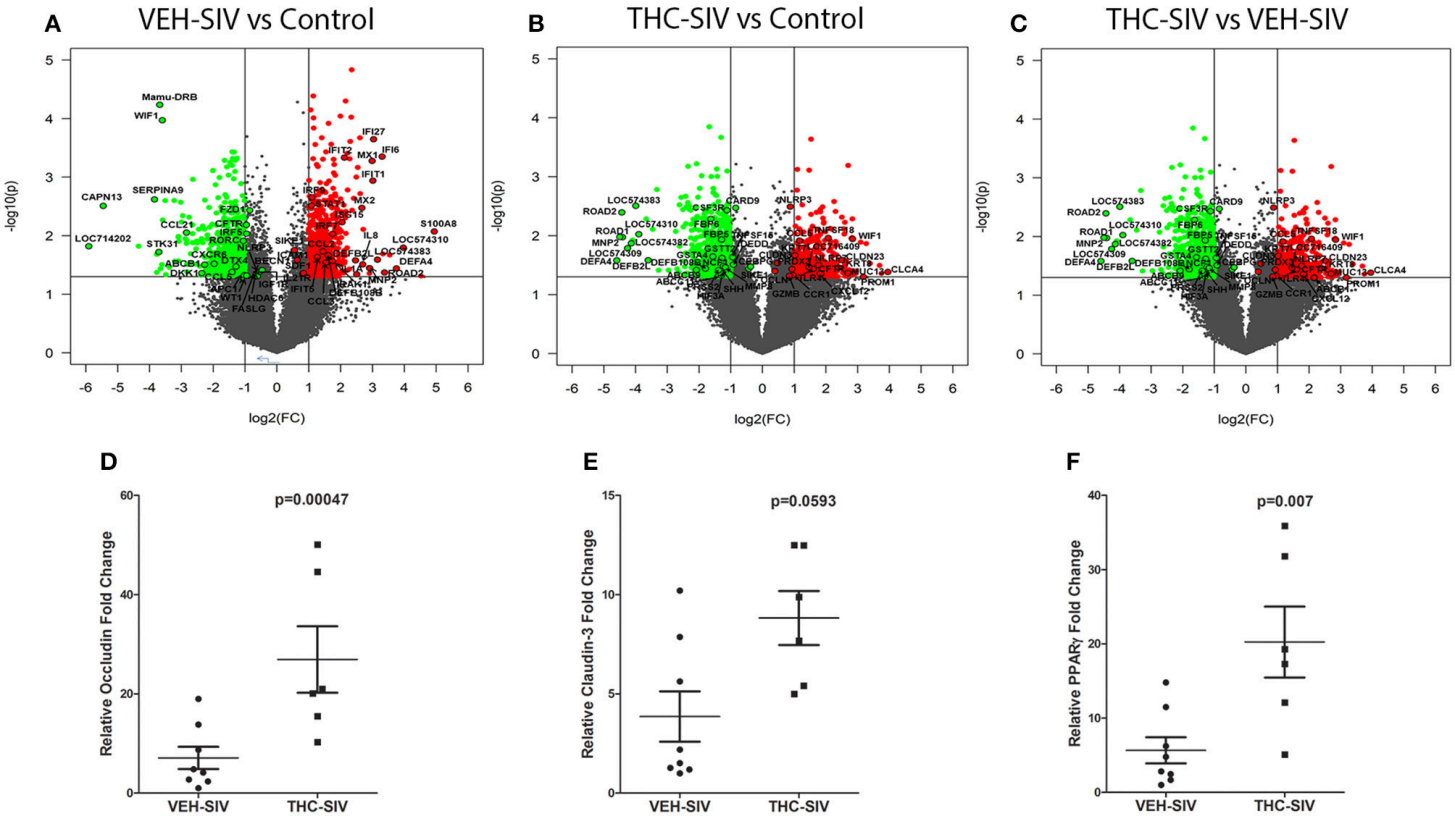
Changes in mRNA expression in the whole colon tissue of chronically SIV-infected rhesus macaques administered vehicle (VEH) or delta-9-tetrahydrocannabinol (Δ^9^-THC). Volcano plot shows the relationship between fold-change (X-axis) and statistical significance (Y-axis) of differentially expressed mRNAs in VEH/SIV **(A)** and THC/SIV **(B)** rhesus macaques relative to controls and in THC/SIV relative to VEH/SIV rhesus macaques **(C)**. The vertical lines in **(A–C)** correspond to 2.0-fold up and down, respectively, and the horizontal line represents *p* ≤ 0.05. The negative log of statistical significance (*p-value*) (base 10) is plotted on the Y-axis, and the log of the fold change base (base 2) is plotted on the X-axis. All differentially expressed mRNAs listed in Supplementary Tables 5–10 are listed and highlighted with a black outer border in **(A–C)**. RT-qPCR confirmation of OCLN **(D)**, CLDN3 **(E)**, and PPARγ **(F)** mRNA expression in the jejunal epithelium of THC/SIV compared to VEH/SIV rhesus macaques. The RT-qPCR analysis was performed twice, and the data were analyzed using the non-parametric Wilcoxon's rank sum test for independent samples. The error bars represent standard error of mean fold change within each group.

To better understand the mechanism of Δ^9^-THC action, we also performed genome-wide transcriptome analysis in the colons of a subset of animals (Table 1) from all three groups. Tables S5, S6 in Supplementary Material show a select list of genes with strong relevance to intestinal inflammation that exhibited upregulation or downregulation, respectively, in VEH/SIV compared to control rhesus macaques. Although the majority of the genes listed in Tables S5, S6 in Supplementary Material showed more than 2-fold change, we included several genes with fold change ranging from 1.5 to 2.0 due to their importance to colonic homeostasis and immune function. Relative to control rhesus macaques, 919 and 1,047 genes were up and downregulated, respectively, in VEH/SIV rhesus macaques. Genes encoding inflammatory molecules [*S100A8, IL-8* (both neutrophil chemoattractant and activator)*, CCL2* (monocyte chemoattractant)*, ICAM1* (leukocyte trans-endothelial migration) and *IL-1*α, considered the “intestinal epithelial danger signal”] (34) were significantly upregulated in VEH/SIV rhesus macaques (Figure 2A). Additionally, *IRAK1*, a key signal transducing kinase in the IL-1 receptor and TLR signaling pathway also showed significant upregulation. Consistent with *IL-1*α upregulation, mRNA encoding seven defensin proteins, *MNP2* (defensin-alpha 2)*, ROAD2* (oral defensin-alpha 2)*, LOC574310* (defensin-alpha 3 precursor)*, DEFA4* (defensin-alpha 4)*, LOC574383* (defensin-alpha 6 precursor)*, DEFB2L* (defensing-beta 2-like), *and DEFB108B* (defensin-beta 108B) showed significantly elevated expression. In addition, upregulation of eight interferon-stimulated genes (ISGs) *IFI6, IFI27, IFIT1, IFIT2, IFI5, ISG15, MX1* (anti-viral)*, MX2* (anti-HIV/SIV), and their inducing transcription factors *IRF7, IRF9*, and *STAT1*, suggests a mechanism to maintain the anti-viral state (Figure 2A). Upregulation of *CCL3 (MIP1*α*), SDF-1 (CXCL12)* (both anti-viral/HIV) (35, 36)*, and SIKE* (suppressor of IKK-epsilon), an inhibitor of interferon-stimulated response elements and the *IFN-beta* promoter is consistent with the host's response to reduce viral replication and the resultant inflammatory response. *CCL2*, a chemokine strongly linked to high levels of immune activation and inflammation in HIV-infected patients and recently identified as a target to reduce immune activation (37) was also significantly upregulated.

Notable downregulated genes included the anti-HIV chemokine (*CCL5*/*RANTES*) (36), anti-microbial chemokine (*CCL21*), Wnt signaling pathway inhibitors (*WIF1, DKK1*, and *APC)*, ATP-binding cassette (ABC) transporter *ABCB1*, ion transport channel *CFTR*, and the central autophagy regulator *BECN1* (Figure 2A).

### Δ^9^-THC Prevented Colonic Anti-microbial Defensin Upregulation and Enhanced Expression of Genes Associated With Maintenance of Epithelial Barrier Integrity

Relative to the control group, 555 and 985 genes were significantly up and downregulated, respectively, in colons of THC/SIV rhesus macaques. This implies a ~1.7-fold decrease in the number of upregulated genes in THC/SIV (Figure 2B) compared to VEH/SIV rhesus macaques (Figure 2A). Like VEH/SIV rhesus macaques, the pro-inflammatory and neutrophil chemotactic genes *S100A8* and *IL8* were significantly increased in THC/SIV rhesus macaques. However, 50% fewer ISGs (*IFITB, IFI27, IFIT1, and MX1 (anti-viral)* (*n* = 4) and their activating transcription factors (*STAT1 and IRF9*) were upregulated in THC/SIV (*n* = 4) rhesus macaques (Table S7 in Supplementary Material). Interestingly, *IRF2*, a competitive inhibitor of *IRF1*-mediated transcriptional activation of interferons alpha and beta and ISGs showed increased expression only in THC/SIV rhesus macaques. Another notable finding was the upregulation of *TNFSF18/GITR* (38) and IL33 (34, 39, 40), two key genes associated with T-regulatory and innate lymphoid cell function in the intestine (Figure 2B).

Different from VEH/SIV rhesus macaques, the expression of the anti-HIV chemokine *CCL3 (MIP1*α*)* (36) was significantly increased in THC/SIV rhesus macaques (3.6 vs. 2.2). Additionally, a second anti-HIV chemokine *CCL8 (MCP-2)* (41), which was not detected in VEH/SIV rhesus macaques showed markedly elevated (~4.7-fold) expression in THC/SIV rhesus macaques (Figure 2B). The most noticeable finding was the complete absence of anti-microbial defensin upregulation in the colons of THC/SIV rhesus macaques (normalized signal intensity same as controls) despite the significant upregulation of seven defensin encoding genes (range- 3 to 15-fold) in VEH/SIV rhesus macaques (Figure 2A). Other important genes that were significantly downregulated in THC/SIV rhesus macaques included *CXCR5, IL2, STAT5A, XAF1* (apoptosis inducer), *and PRSS2* (Intestinal defensin processing serine protease) (42) (Table S8 in Supplementary Material). Overall, relative to controls, THC/SIV rhesus macaques showed increased expression of anti-HIV/SIV chemokines, fewer ISGs and failed to upregulate intestinal anti-microbial defensin encoding genes. The decreased expression of *PRSS2* may partially explain the absence of defensin upregulation in colons of THC/SIV rhesus macaques.

The anti-inflammatory effects of Δ^9^-THC became more prominent when we directly compared VEH/SIV and THC/SIV rhesus macaques. Interestingly, ~8 *alpha* and 2 *beta-defensins, MMP8* (both produced by neutrophils and epithelial cells) (12, 43), *SOCS3* (44), and *C/EBP*γ (45) showed significantly reduced expression (Figure 2C) suggesting relatively diminished inflammatory responses in colons of THC/SIV rhesus macaques (Table S9 in Supplementary Material). In contrast, genes linked to pathways that control intestinal epithelial proliferation, differentiation and function such as *WIF1* (a negative regulator of the Wnt signaling pathway)*, KRT*8 (46) and *CFTR* (chloride ion transport) showed significantly increased expression (Table S10 in Supplementary Material). The anti-microbial chemokine *CXCL12*, anti-HIV/SIV chemokine *CCL5* and its receptor *CCR1* (41), *GZMB* (cytotoxic T cell-associated serine esterase I)*, NLRP3* (inflammasome)*, PRDX3* (mitochondrial anti-oxidant) showed enhanced expression in THC/SIV rhesus macaques (Figure 2C). Importantly, expression of tight junction (TJ) proteins Claudin-3 (*CLDN3*) and Occludin (*OCLN*), *PROM1*, a marker of intestinal epithelial regeneration (47) and *MUC13*, an inhibitor of intestinal inflammation and epithelial apoptosis (48) were significantly enhanced in THC/SIV rhesus macaques (Figure 2C). Apart from the colon, we also confirmed upregulation of *OCLN* and *CLDN3* in the jejunal epithelium (JE) of THC/SIV rhesus macaques (Figures 2D,E) suggesting that Δ^9^-THC preserves tight junction protein expression in both small and large intestine. While *PPAR*γ, an important anti-dysbiotic transcription factor (49) that is downregulated in SIV infection (18), failed to achieve statistical significance in microarray analysis, focused RT-qPCR analysis confirmed statistically significant upregulation in JE of THC/SIV rhesus macaques (Figure 2F). Although statistically non-significant, relatively lower plasma lipopolysaccharide binding protein levels were detected in THC/SIV (*n* = 5) (average 46 ng/ml) compared to VEH/SIV rhesus macaques (*n* = 4) (average 25 ng/ml) (Figure S3 in Supplementary Material) at 180 days post infection.

### MMP8 Is a Direct Target of miR-204 and its Protein Expression Is Significantly Decreased in Colons of THC/SIV Rhesus Macaques

**Figure 3:**
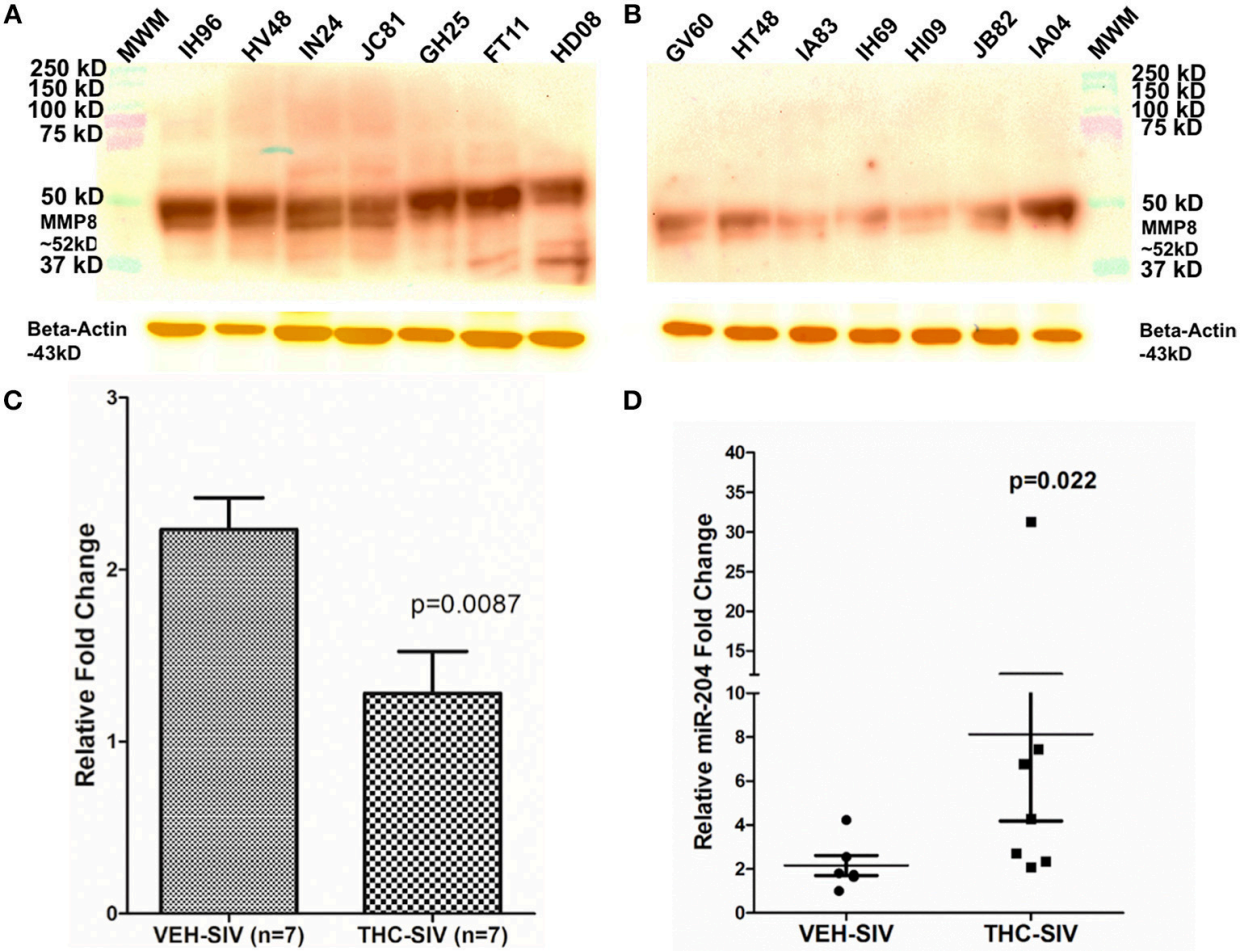
MMP8 protein expression is significantly reduced in whole colon tissue of THC/SIV rhesus macaques. Representative western blot **(A,B)** and quantification **(C)** showing a reduction in protein expression of MMP8 (~52 kD) in whole colon tissue of THC/SIV rhesus macaques. Immunoprecipitation/Western blotting was performed twice with the same samples. RT-qPCR confirmed significant upregulation of miR-204, a miRNA computationally predicted to directly target MMP8, in colonic epithelium of THC/SIV rhesus macaques **(D)**. The RT-qPCR analysis was performed twice with the same samples. Immunoprecipitation/western blot densitometry data **(C)** was analyzed using Mann-Whitney *U*-test. RT-qPCR data were analyzed using the non-parametric Wilcoxon's rank sum test for independent samples. The error bars represent standard error of mean fold change within each group. MWM- Molecular weight marker.

Unlike our previous studies (18), a genome-wide integrative miRNA-mRNA analysis was not performed as the use of whole colon tissue made it impossible to confirm if the differential expression of a specific miRNA and its mRNA target occurred in the same cell type. Nevertheless, the miR-204-*MMP8* pairing was characterized as the enhanced *MMP8* expression has been confirmed in the colonic epithelium (CE) and lamina propria neutrophils of IBD patients (12). Consistent with findings in IBD patients, we detected significantly elevated *MMP8* protein expression in colons of VEH/SIV (Figure 3A) but not in THC/SIV rhesus macaques (Figures 3B,C). Accordingly, we detected significantly high miR-204 expression in colons (Figures 1C,F) of THC/SIV rhesus macaques and confirmed its high expression specifically in CE (Figure 3D) compared to VEH/SIV rhesus macaques. Although miR-204 showed ~1.5-fold increase in whole colon tissue (Figures 1C,F), its expression specifically in CE of THC/SIV rhesus macaques was increased by ~8.5-fold (Figure 3D). As miR-204 is also downregulated during intestinal inflammation in the human (50), we hypothesized that this would facilitate increased *MMP8* expression in CE of THC/SIV rhesus macaques.

**Figure 4:**
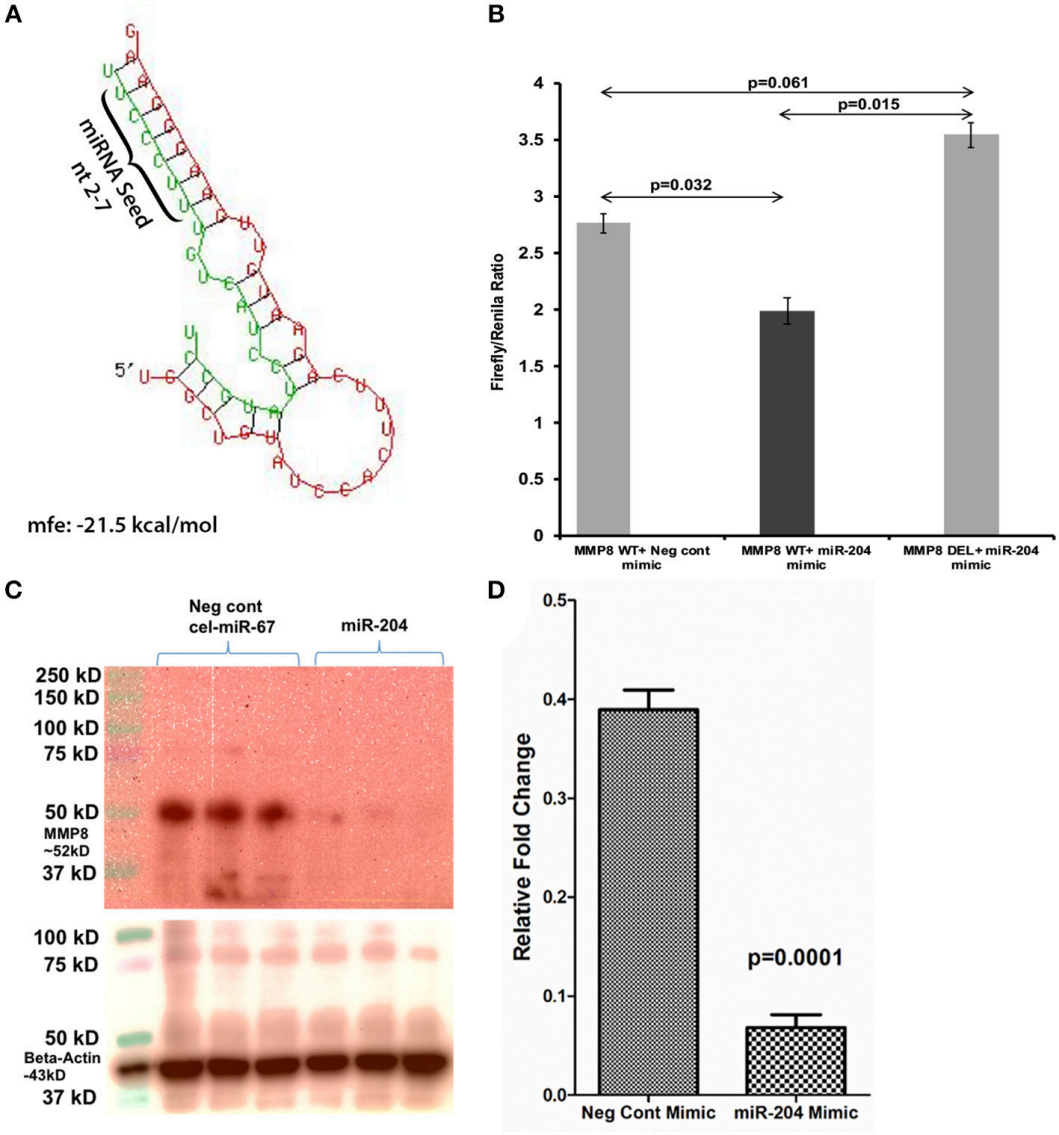
MMP8 is a direct target of miR-204. Luciferase reporter vectors containing a single highly conserved miR-204 **(A)** binding site (seed nucleotide region) on the rhesus macaque MMP8 mRNA 3′ UTR or the corresponding construct with the binding sites deleted were co-transfected into HEK293 cells with 30 nM miR-204 or Negative control mimic. *Firefly* and *Renilla* luciferase activities were detected using the Dual-Glo luciferase assay system 96 h after transfection. Note significantly reduced Firefly/*Renilla* ratios following co-transfection of miR-204 mimic with a pmirGLO vector containing wild-type (WT) miRNA binding sties **(B)**. Luciferase reporter assays were performed thrice in six replicate wells. Representative Immunoprecipitation/western blot (Triplicate wells) **(C)** and quantification **(D)** showing reduction in MMP8 protein expression (~52 kD) 96 h post transfection of HEK293 cells in triplicate wells with negative control (cel-miR-67) or miR-204 mimics. miR-204 overexpression followed by immunoprecipitation/western blot was performed twice in triplicate wells. *Firefly/Renilla* ratios were analyzed using one-way ANOVA followed by Benjamini-Hochberg *post hoc* test (*p* < 0.05). Western blot densitometry data were analyzed using unpaired “*t*”-test. A *p*-value of ≤0.05 was considered significant.

The 3′ UTR of the rhesus macaque *MMP8* mRNA contains a single proximal miR-204 binding site (Nucleotide positions 41-47—Ensembl ID: ENST00000236826.3) that is highly conserved across eight different mammalian species (19). An *in-silico* miRNA-mRNA duplex analysis using RNAhybrid (51) yielded a significantly low minimum free energy (mfe< −20 kcal/mol), which confirmed *MMP8* as a predicted target of miR-204 (Figure 4A). Luciferase reporter assays confirmed the ability of miR-204 to directly bind the 3′ UTR of *MMP8* (34% reduction in *firefly/renilla* ratios), and potentially downregulate its protein expression (Figure 4B). Next, we moderately overexpressed miR-204 (HEK293 cells) in triplicate wells and detected a significant reduction in *MMP8* protein levels (Figures 4C,D) 72 h post transfection.

### Chronic Δ^9^-THC Administration Markedly Reduced Collagen Deposition in Lymph Nodes of Chronically SIV-Infected Rhesus Macaques

**Figure 5:**
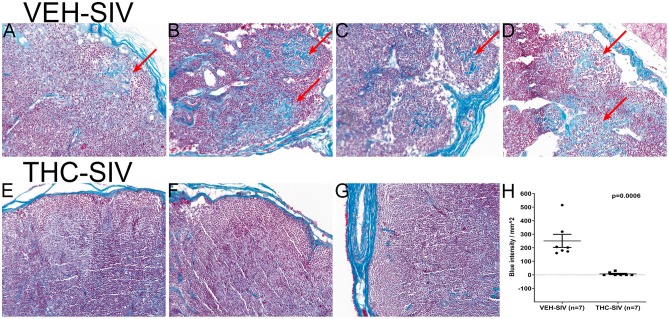
VEH/SIV rhesus macaques have significantly high amounts of collagen deposition in lymph node paracortex and B-cell follicles **(A–D)** (red arrows) compared to THC/SIV rhesus macaques **(E–G)**. Note blue staining collagen (red arrow) (Gomori one-step trichrome stain) in **(A–D)**. Image quantification confirmed high collagen deposition in lymph nodes of VEH/SIV rhesus macaques **(H)**.

To verify if the anti-inflammatory effects of Δ^9^-THC extended beyond the intestine, we focused on lymph nodes, where HIV/SIV induced chronic inflammation has been proposed to promote collagen deposition, thereby impeding CD4^+^ T cell restoration and generation of immune responses (52). Consequently, we stained axillary lymph node sections from 7 VEH/SIV and 7 THC/SIV rhesus macaques following Gomori's one-step trichrome staining method (24) for the detection of collagen deposition. Despite the presence of lymphoid hyperplasia in both groups, collagen deposition (blue staining in Figures 5A–D) extending into the paracortex and B-cell follicular zone was prominent in four out of seven VEH/SIV rhesus macaques (Figures 5A–D). Interestingly, collagen staining in the same lymph node areas from all seven THC/SIV rhesus macaques (Figures 5E–G and Figure S4 in Supplementary Material) was minimal. Image quantification confirmed high collagen deposition in lymph nodes of VEH/SIV rhesus macaques (Figure 5H).

### Δ^9^-THC Significantly Reduced the Expression of T Cell Proliferation/Activation Markers and PD-1 in the Intestine as Early as 14 Days Post SIV Infection Compared to the VEH/SIV Group

**Figure 6:**
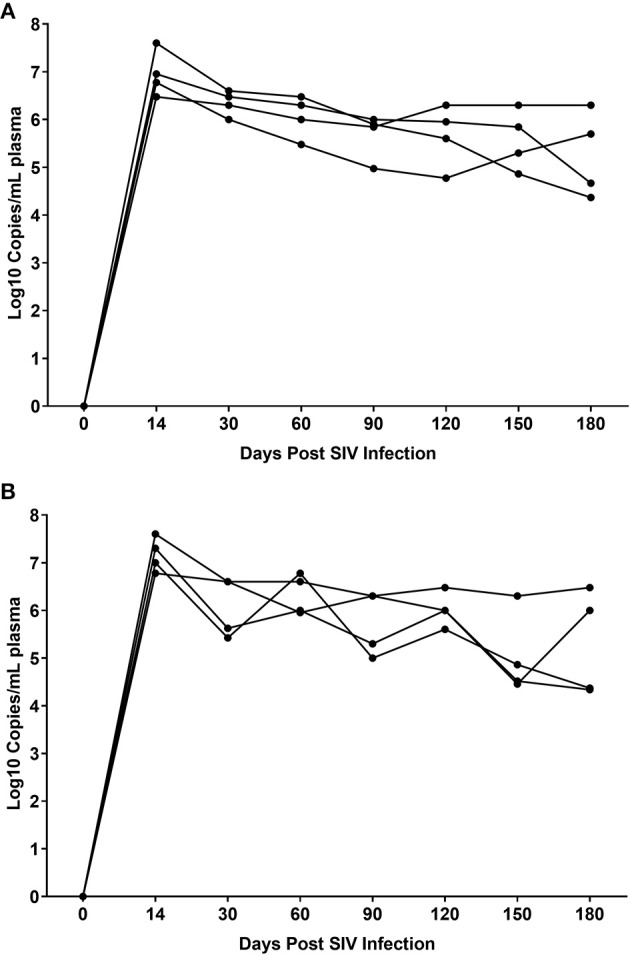
Plasma viral loads in VEH/SIV **(A)** (*n* = 4) and THC/SIV **(B)** (*n* = 4) rhesus macaques through the course of SIV infection. Viral load measurements were performed once using 1-step RT-qPCR using four individual animals as biological replicates per group. Differences between groups at each post infection time point were analyzed using the Mann-Whitney U test employing the Prism v5 software (GraphPad software). Note the similarity in peak plasma viremia in both groups at 14 days post SIV infection. No statistical differences in plasma viral loads were detected between the two groups at all post infection time points.

**Figure 7:**
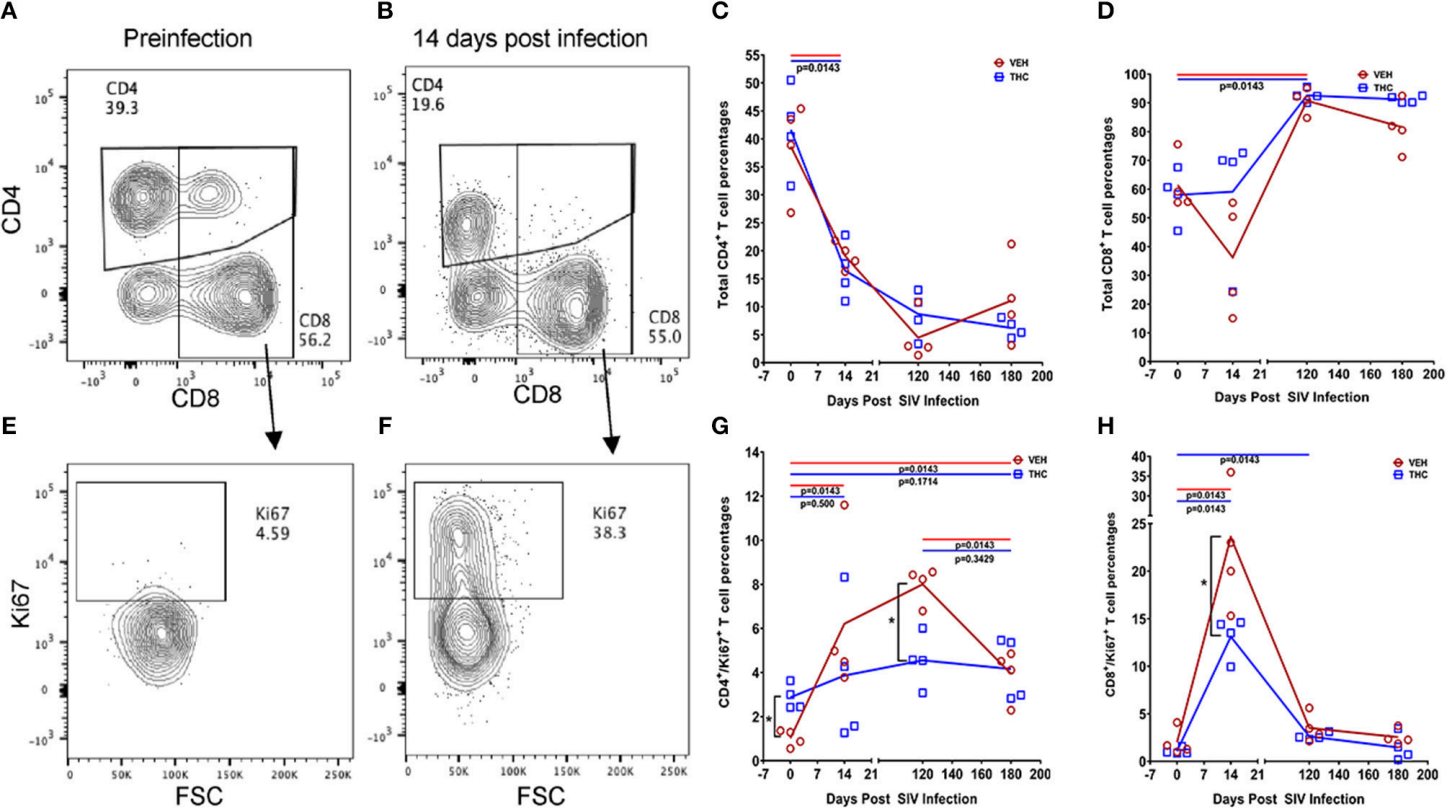
Delta-9-tetrahydrocannabinol (Δ^9^-THC) administration regulates intestinal T cell proliferation/activation in SIV infection. A representative contour plot shows single positive CD4^+^ and double positive CD4^+^/CD8^+^ T cell depletion in the intestine at 14 days post infection in a VEH/SIV rhesus macaque **(A,B)**. All acquired cells were first gated on singlets, followed by CD45^+^ and live cells. All live cells were further gated for CD3^+^ and CD20^+^ cells, where T cells were defined by gating for CD3^+^ T-cells. Finally, CD3^+^ T cells were analyzed for the presence of CD4^+^ and CD8^+^ phenotype. Total CD4^+^ T cell percentages are significantly reduced in intestines of both VEH/SIV and THC/SIV rhesus macaques as early as 14 days post-infection and remained low throughout the study period **(C)**. In contrast, total CD8^+^ T cell percentages were significantly increased in both VEH/SIV and THC/SIV rhesus macaques **(D)** at 120 days post-SIV infection. Contour plot shows CD8^+^ T cell proliferation/activation based on high Ki67 expression at 14 days post-SIV infection compared to the preinfection time point in a VEH/SIV rhesus macaque **(E,F)**. Within the gated CD4^+^ T cell population, note the significantly attenuated CD4^+^ T cell proliferation based on Ki67 expression in THC/SIV but not VEH/SIV rhesus macaques **(G)** at 14, 120-, and 180-days post-SIV infection compared to their respective preinfection levels. Similarly, Δ^9^-THC significantly attenuated CD8^+^ T cell proliferation/activation associated with peak viral replication at 14 days post-SIV infection compared to VEH/SIV rhesus macaques **(H)**. Open red circles- VEH/SIV, Open blue squares- THC/SIV. Horizontal red and blue lines with *P*-values denote comparisons between time points within the VEH/SIV and THC/SIV groups, respectively. Left black brackets with an asterisk indicate statistical significance (*p* = 0.0143) between the VEH/SIV and THC/SIV groups for a given time point. Connecting blue and red lines **(C,D,G,H)** denote mean values of the respective population in the THC/SIV and VEH/SIV groups, respectively. Flow cytometry analysis to determine the effect of Δ^9^-THC on total CD4^+^ and CD8^+^ T cells and Ki67 expression in SIV-infected rhesus macaques was performed once with four individual animals as biological replicates per group. Flow cytometry data were analyzed using linear mixed models with immune-marker outcomes being dependent variables, and treatment status (VEH vs. Δ^9^-THC) and days since the start of treatment (0, 14, 120, 180) being independent variables with fixed effects. Differences between two time points were analyzed using the Mann-Whitney *U*-test employing the Prism v5 software (GraphPad software). A *p*-value of ≤0.05 was considered significant.

While THC effectively inhibited pro-inflammatory gene and miRNA expression, it was very intriguing to find out if these modulations were accompanied by alterations in plasma/tissue viral loads and expression of T cell proliferation/activation markers, including PD-1 in the intestine. No differences in plasma viral loads were detected between VEH/SIV and THC/SIV rhesus macaques through the course of SIV infection (Figures 6A,B). CD4^+^ T cells were markedly depleted in the intestines at 14 days post-infection (Figures 7A,B) and the depletion persisted through 180 days post-infection in both groups (Figure 7C). Concurrently, CD8^+^ T cell percentages increased at 120- and 180-days post-infection (Figure 7D). We next examined intracellular expression levels of Ki67 (Figures 7E,F) to assess the degree of T cell proliferation/activation in both groups. A significant difference (*p* ≤ 0.05) for days post infection and treatment status (VEH vs. Δ^9^-THC) was detected for CD4^+^/CD8^+^/Ki67^+^ measurements. Within the surviving intestinal CD4^+^ T cell population significant differences were detected between the two groups. Interestingly, THC/SIV rhesus macaques had a significantly (*p* ≤ 0.05) higher percentage of CD4^+^/Ki67^+^ T cells than VEH/SIV rhesus macaques before SIV infection. However, after SIV infection the percentage of CD4^+^/Ki67^+^ T cells (connecting blue line in Figure 7G) in THC/SIV rhesus macaques at 14, 120-, and 180-days post-infection did not differ from preinfection levels. In contrast, the percentage of CD4^+^/Ki67^+^ cells in VEH/SIV rhesus macaques showed a statistically significant increase from 14 until 120 days post-infection (connecting red line in Figure 7G) compared to the pre-infection time point. Similarly, at 14 days post infection, the percentage of CD8^+^/Ki67^+^ cells were massively increased in VEH/SIV compared to pre-infection levels (Figure 7H). Even though a statistically significant increase in the percentage of CD8^+^/Ki67^+^ cells was detected in THC/SIV rhesus macaques, the magnitude of this increase was significantly lower compared to the VEH/SIV group.

**Figure 8:**
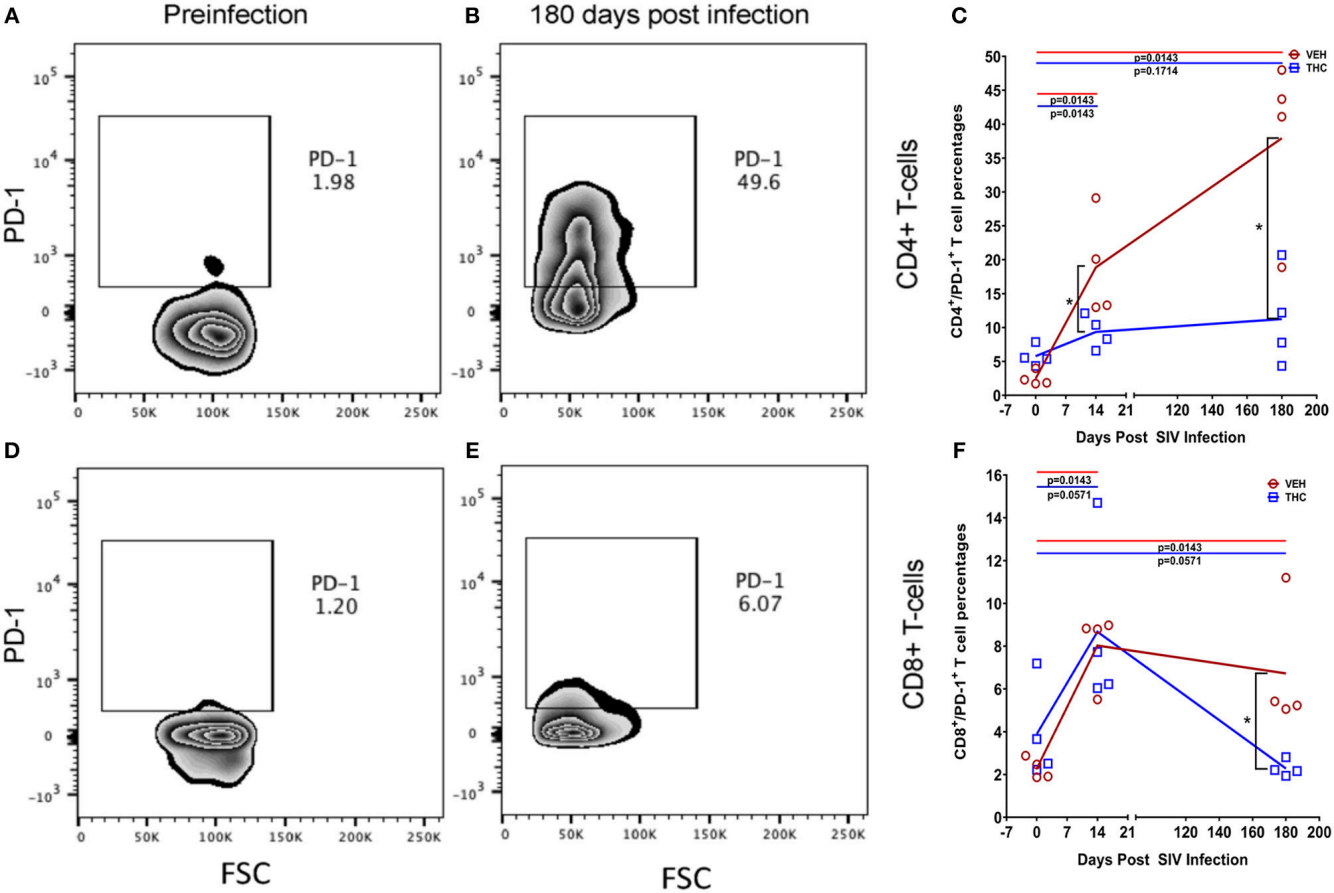
Delta-9-tetrahydrocannabinol (Δ^9^-THC) administration inhibited PD-1 expression in intestinal T cells after SIV infection. Zebra plot shows significantly high expression of programmed death 1 (PD-1) by intestinal CD4^+^ T cells in a VEH/SIV rhesus macaque at 180 days post infection compared to preinfection time point **(A,B)**. In contrast, PD1 expression in intestinal CD4^+^ T cells is significantly reduced in THC/SIV rhesus macaques at 14- and 180-days post-SIV infection compared to VEH/SIV rhesus macaques **(C)**. Zebra plot shows PD-1 expression on intestinal CD8^+^ T cells of a VEH/SIV rhesus macaque at 180 days post-infection **(D,E)**. Like CD4^+^ T cells, the percentage of PD-1 expressing CD8^+^ T cells out of total CD8^+^ T cells was also significantly reduced in THC/SIV rhesus macaques at 180 days post-SIV infection compared to VEH/SIV rhesus macaques **(F)**. Open red circles- VEH/SIV, Open blue squares- THC/SIV. Horizontal red and blue lines with *P*-values denote comparisons between time points within the VEH/SIV and THC/SIV groups, respectively. Left black brackets with an asterisk indicate statistical significance (*p* = 0.0143) between the VEH/SIV and THC/SIV groups for a given time point. Connecting blue and red lines **(C,F)** denote mean values of the respective population in the THC/SIV and VEH/SIV groups, respectively. Flow cytometry analysis to determine the effect of Δ^9^-THC on PD-1 expression in intestinal CD4^+^ and CD8^+^ T cells of SIV-infected rhesus macaques was performed once with four individual animals as biological replicates per group. Flow cytometry data were analyzed using linear mixed models with immune-marker outcomes being dependent variables, and treatment status (VEH vs. Δ^9^-THC) and days since the start of treatment (0, 14, 120, 180) being independent variables with fixed effects. Differences between two time points were analyzed using the Mann-Whitney *U*-test employing the Prism v5 software (GraphPad software). A *p*-value of ≤0.05 was considered significant.

**Figure 9:**
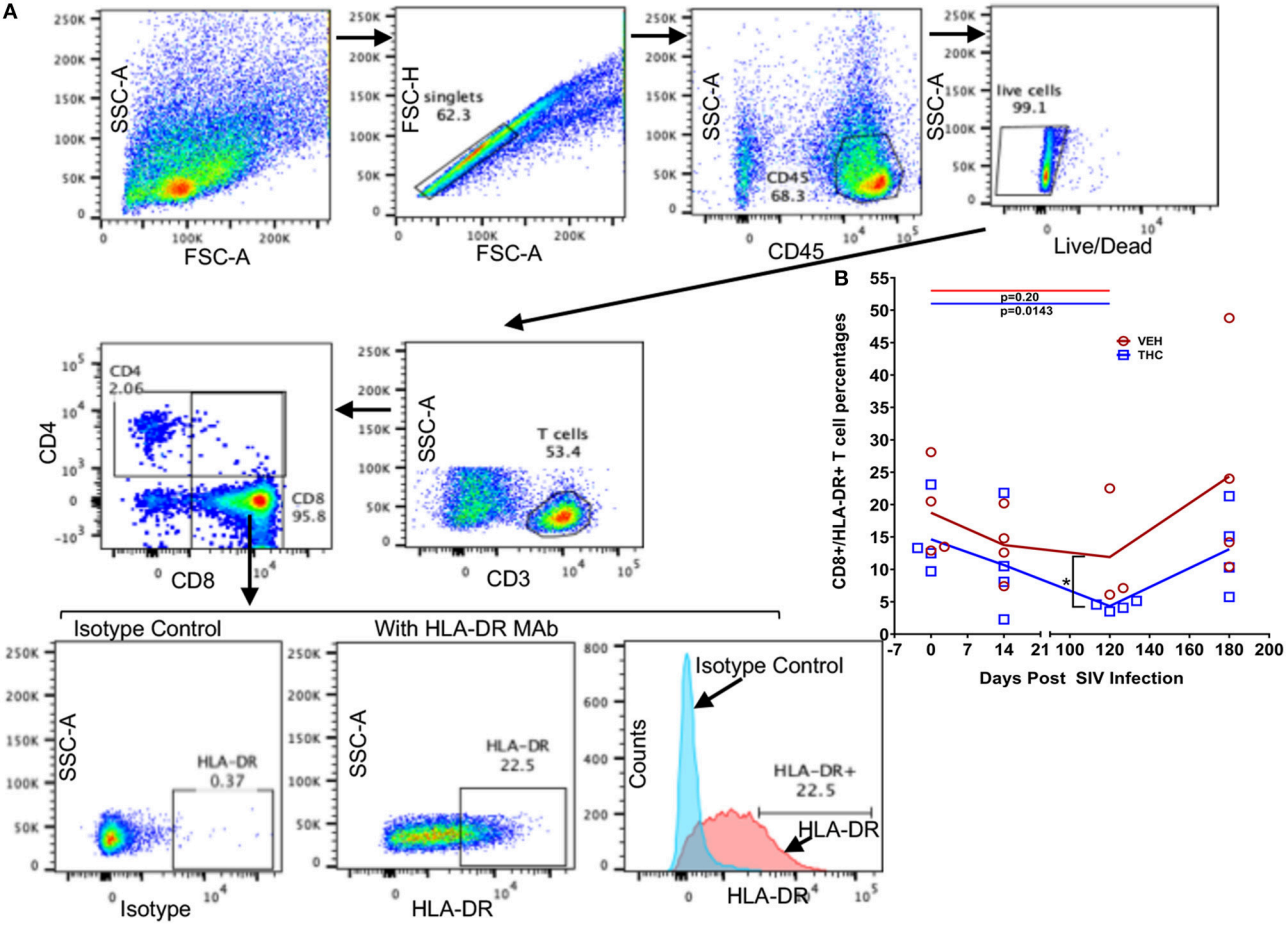
Delta-9-tetrahydrocannabinol (Δ^9^-THC) administration inhibited HLA-DR expression in intestinal CD8^+^ T cells after SIV infection. Gating strategy for HLA-DR expression from a representative VEH/SIV rhesus macaque **(A)**. Cells were gated first on singlets, CD45 (lymphocyte common antigen), followed by live cells, and then on CD3^+^ T cells. T-cells were further gated on CD3^+^CD4^+^ and CD3^+^CD8^+^ T cell subsets. The percentages of total HLA-DR expression in CD8^+^ T cells are shown in the bottom row where the positive HLA-DR expression was compared to the isotype control both in the dot plots and histograms. **(B)** THC/SIV rhesus macaques showed reduced percentage of CD8^+^ T cells expressing the late activation marker HLA-DR at 120 days post SIV compared to VEH/SIV rhesus macaques. Note that data from only three VEH/SIV rhesus macaques were available for the 120 days post-infection time point as LPL yields from one animal were insufficient for flow cytometry. Open red circles- VEH/SIV, Open blue squares- THC/SIV. Horizontal red and blue lines with *P*-values denote comparisons between time points within the VEH/SIV and THC/SIV groups, respectively. Left black brackets with an asterisk indicate statistical significance (*p* = 0.0286) between the VEH/SIV and THC/SIV groups for a given time point. Connecting blue and red lines **(B)** denote mean values of the respective population in the THC/SIV and VEH/SIV groups, respectively. Flow cytometry analysis to determine the effect of Δ^9^-THC on HLA-DR expression in intestinal CD8^+^ T cells of SIV-infected rhesus macaques was performed once with three to four individual animals as biological replicates per group. Flow cytometry data were analyzed using linear mixed models with immune-marker outcomes being dependent variables, and treatment status (VEH vs. Δ^9^-THC) and days since the start of treatment (0, 14, 120, 180) being independent variables with fixed effects. Differences between two time points were analyzed using the Mann-Whitney *U*-test employing the Prism v5 software (GraphPad software). A *p*-value of ≤0.05 was considered significant.

**Figure 10:**
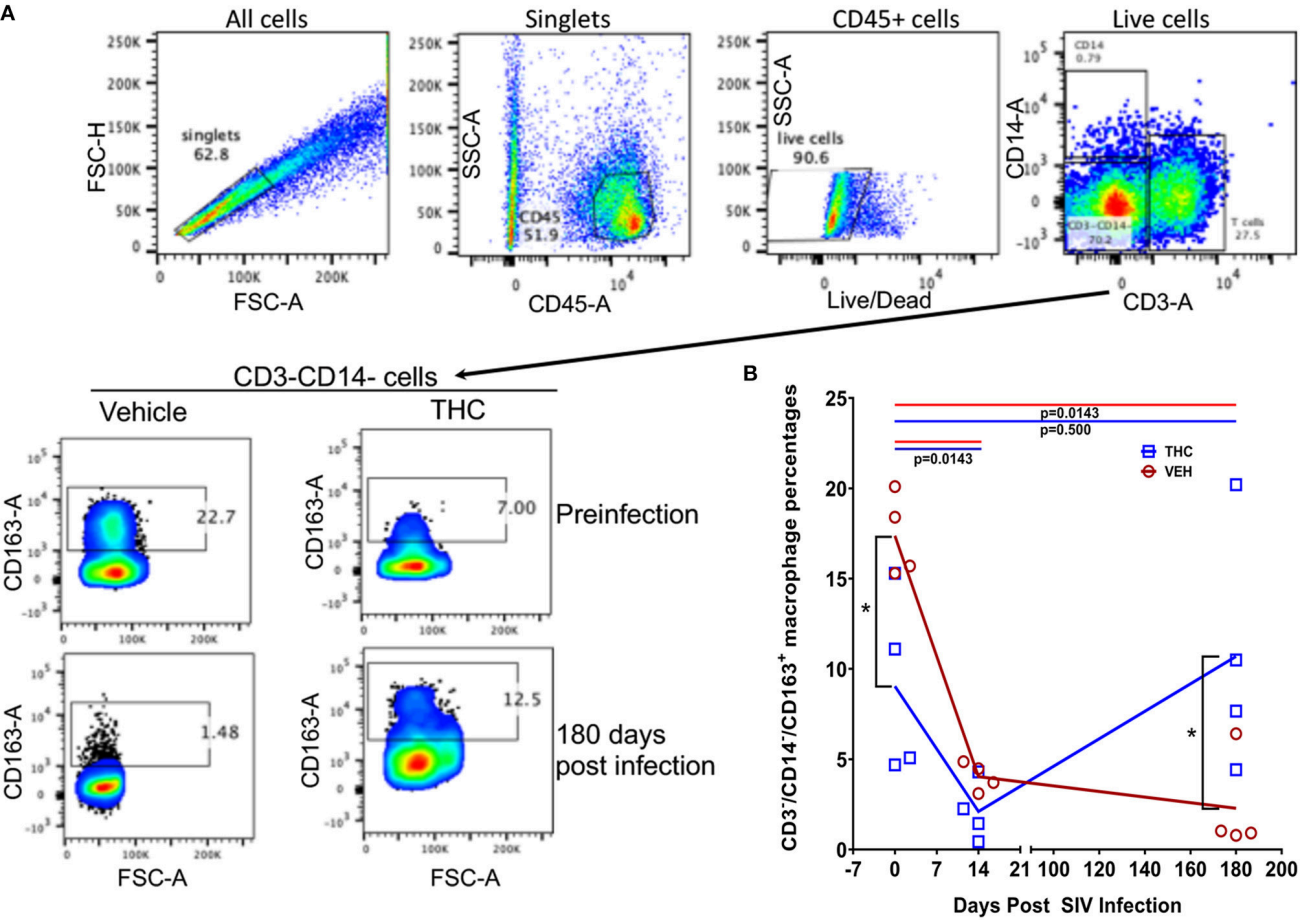
Delta-9-tetrahydrocannabinol (Δ^9^-THC) administration increased the percentage of CD163 expressing anti-inflammatory macrophages in intestine after SIV infection. To quantify CD163^+^ anti-inflammatory macrophages, cells were gated first on singlets, CD45^+^ cells, followed by live cells and then on CD3^−^CD14^−−^ cell subsets to quantify CD163^+^ cells **(A)**. Frequencies of CD163^+^ cells at preinfection and 180 days post-infection are shown from a representative THC/SIV and VEH/SIV rhesus macaque **(A)**. The percentages of the total gated population are shown in each box of the plot. Note that the THC/SIV infected animal had significantly higher CD163^+^ cells compared to VEH/SIV infected animal at 180 days post-SIV infection **(A)**. Open red circles- VEH/SIV, Open blue squares- THC/SIV. Horizontal red and blue lines with *P*-values denote comparisons between time points within the VEH/SIV and THC/SIV groups, respectively. Left black brackets with an asterisk indicate statistical significance (*p* = 0.0286) between the VEH/SIV and THC/SIV groups for a given time point. Connecting blue and red lines **(B)** denote mean values of the respective population in the THC/SIV and VEH/SIV groups, respectively. Flow cytometry analysis to determine the effect of Δ^9^-THC on CD163 expression in intestinal macrophages of SIV-infected rhesus macaques was performed once with four individual animals as biological replicates per group. Flow cytometry data were analyzed using linear mixed models with immune-marker outcomes being dependent variables, and treatment status (VEH vs. Δ^9^-THC) and days since the start of treatment (0, 14, 150, 180) being dependent variables with fixed effects. Differences between two time points were analyzed using the Mann-Whitney *U*-test employing the Prism v5 software (GraphPad software). A *p*-value of ≤0.05 was considered significant.

We next examined the expression levels of PD-1, a molecule associated with T cell activation/exhaustion and HIV/SIV disease progression and persistence (53) in CD4^+^ and CD8^+^ T cells (Figures 8A,B). A significant difference (*p* ≤ 0.05) for days post infection and treatment status (VEH vs. Δ^9^-THC) was detected for CD4^+^/PD-1^+^ measurements. While the percentage of CD4^+^/PD-1^+^ cells steadily increased in the intestines of VEH/SIV rhesus macaques, their corresponding percentages in THC/SIV rhesus macaques remained stable from 14 until 180 days post-infection (Figure 8C). In intestinal CD8^+^ T cells (Figures 8D,E), a significantly higher percentage of CD8^+^/PD-1^+^ cells were detected at 14- and 180-days post-infection only in VEH/SIV rhesus macaques compared to the preinfection time point (Figure 8F and Figure S5 in Supplementary Material). At 180 days post infection, VEH/SIV rhesus macaques had significantly higher percentages of CD8^+^/PD-1^+^ cells compared to THC/SIV rhesus macaques (Figure 8F). Due to limited cell availability, we could not assess PD-1 levels at 120- or 150-days post infection. Further, at 120 days post-infection the percentage of CD8^+^ T cells expressing the late T cell activation marker HLA-DR was significantly decreased in the intestines of THC/SIV but not in VEH/SIV rhesus macaques (Figures 9A,B). At 180 days post infection, HLA-DR expressing CD8^+^ T cell percentages increased in both groups but were substantially lower in THC/SIV rhesus macaques (Figure 9B). We next quantified CD163^+^ macrophages that were negative for CD3 and CD14 in intestinal tissues (Figure 10A). Like Ki67 and PD-1, a significant difference (*p* ≤ 0.05) for days post infection and treatment status (VEH vs. Δ^9^-THC) was detected for CD163^+^ measurements. Compared to the preinfection time point, the percentage of CD163^+^ anti-inflammatory macrophages (Figure 10B) (54) was significantly reduced at 180 days post-infection in VEH/SIV but not in THC/SIV rhesus macaques (Figure 10B). Moreover, at 180 days post infection, THC/SIV rhesus macaques had a significantly higher percentage of CD163^+^ anti-inflammatory macrophages (54) in the intestine compared to the VEH/SIV group (Figure 10B).

**Figure 11:**
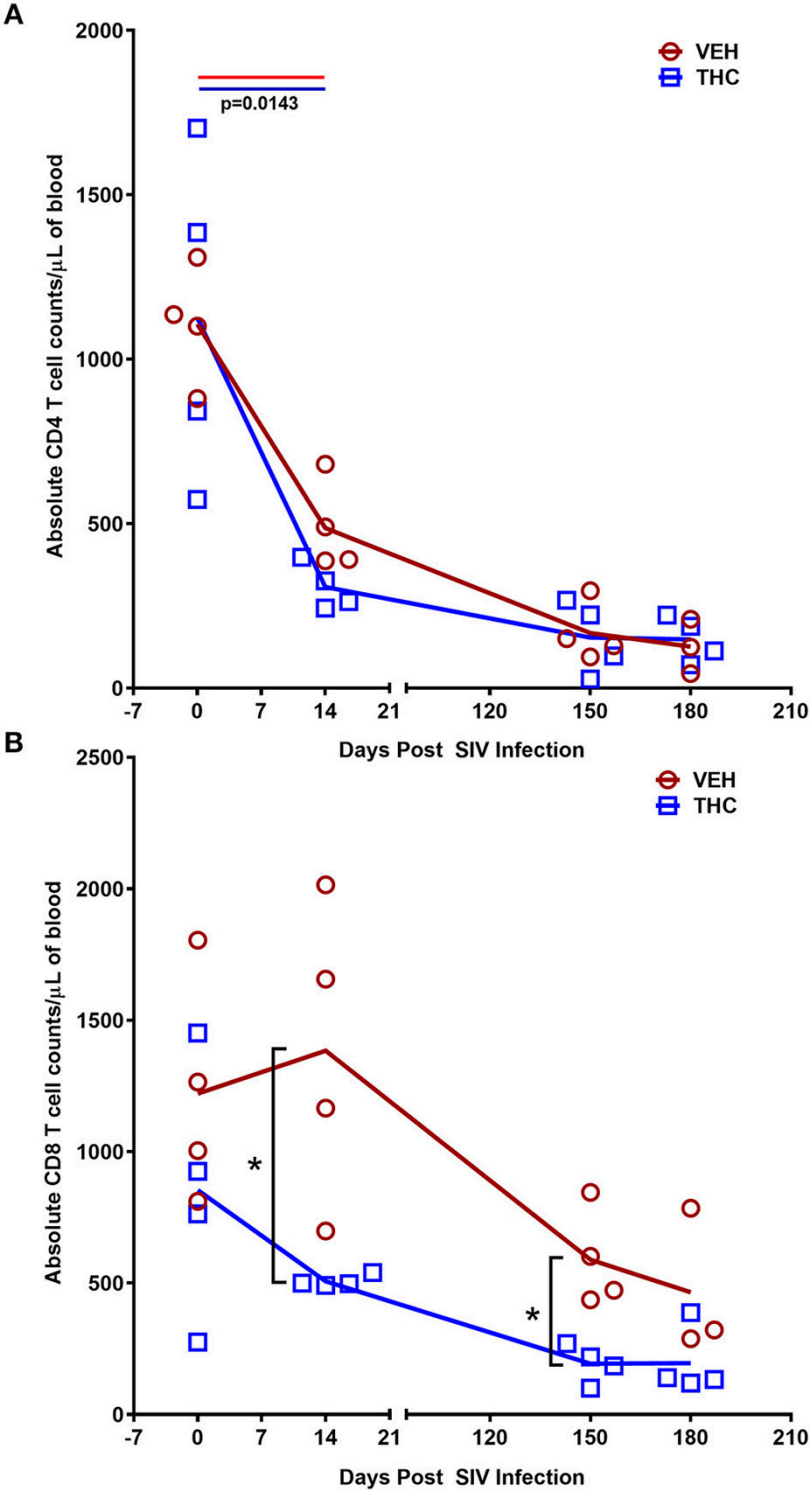
Delta-9-tetrahydrocannabinol (Δ^9^-THC) administration inhibited CD8^+^ T cell expansion in peripheral blood during chronic SIV infection. Absolute counts revealed that Delta-9-Tetrahydrocannabinol (Δ^9^-THC) administration did not prevent depletion of CD4^+^ T cells **(A)** but significantly reduced total CD8^+^ T cell numbers **(B)** in peripheral blood at 14- and 150-days post-SIV infection. Note that CD8^+^ T cell data from only three VEH/SIV rhesus macaques were available at 180 days post-infection. Open red circles- VEH/SIV, Open blue squares- THC/SIV. Horizontal red and blue lines with *P*-values denote comparisons between time points within the VEH/SIV and THC/SIV groups, respectively. Left black brackets with an asterisk indicate statistical significance (*p* = 0.0143) between the VEH/SIV and THC/SIV groups for a given time point. Connecting blue and red lines **(A,B)** denote mean values of the respective population in the THC/SIV and VEH/SIV groups, respectively. Flow cytometry analysis to determine the effect of Δ^9^-THC on peripheral blood T cell dynamics in SIV-infected rhesus macaques was performed once with three to four individual animals as biological replicates per group. Flow cytometry data were analyzed using linear mixed models with immune-marker outcomes being dependent variables, and treatment status (VEH vs. Δ^9^-THC) and days since the start of treatment (0, 14, 150, 180) being dependent variables with fixed effects. Differences between two post-infection time points were analyzed using the Mann-Whitney *U*-test employing the Prism v5 software (GraphPad software). A *p*-value ≤ 0.05 was considered significant.

For peripheral blood CD4^+^ and CD8^+^ T cell percentages, a significant difference (*p* ≤ 0.05) was detected post infection. Like the intestine, both VEH/SIV and THC/SIV rhesus macaques showed significant depletion of total CD4^+^ T cells (absolute counts) in peripheral blood (Figure 11A) at 14 days post-infection that continued to remain low until 180 days post infection. Unlike CD4^+^, the total CD8^+^ T cell numbers were significantly higher at 14- and 150-days post infection in VEH/SIV compared to THC/SIV rhesus macaques (Figure 11B). At 180 days post infection, total CD8^+^ T cell numbers remained high in VEH/SIV rhesus macaques but did not achieve statistical significance (*p* = 0.1143; Figure 11B).

## Discussion

Chronic intestinal inflammation is a common sequela to HIV/SIV replication and persists even in virally suppressed PLWH (8). This inflammation is detrimental as it can disrupt the intestinal epithelial barrier and promote microbial/byproduct translocation and interfere with absorption of cART drugs, both of which contribute to enhanced viral replication, systemic immune activation, HIV associated comorbidities and disease progression (8). Therefore, feasible pharmacological strategies to reduce/inhibit chronic intestinal inflammation are needed to increase cART efficacy and restore GI homeostasis and immune function. In the present study, we demonstrate that differential modulation of miRNA/gene expression and T cell proliferation/activation are potential mechanisms underlying the anti-inflammatory effects of Δ^9^-THC in chronic untreated SIV infection.

Consistent with the anti-inflammatory effects of Δ^9^-THC, fewer miRNAs were differentially expressed in the colons of THC/SIV (*n* = 32) than VEH/SIV (*n* = 52) rhesus macaques, compared to controls. Also, the number of upregulated miRNAs was 2-fold lower in THC/SIV (*n* = 11) than VEH/SIV (*n* = 23) rhesus macaques. The most noteworthy was miR-146b-5p, an LPS-responsive miRNA (31), which was not elevated in THC/SIV rhesus macaques compared to controls. We previously confirmed increased miR-146b-5p expression in the intestinal lamina propria of chronic SIV-infected rhesus macaques and in cultured primary intestinal/bone marrow macrophages following LPS treatment (17). More recently, we reported miR-130a upregulation in CE during chronic SIV infection and its ability to disrupt the epithelial barrier by downregulating PPARγ (18). Interestingly, both miRNAs are upregulated in colons of IBD patients (28, 55) and suggest pro-inflammatory signaling as a driver of its increased expression in the colon of VEH/SIV rhesus macaques. Further, compared to VEH/SIV rhesus macaques, Δ^9^-THC significantly downregulated the expression of pro-inflammatory miR-21, miR-141, and miR-222.

We next compared gene expression and found significantly elevated *SA100A8* and *IL8* expression in both VEH/SIV and THC/SIV rhesus macaques relative to controls. However, significantly fewer interferon-stimulated genes were upregulated in THC/SIV rhesus macaques. Additionally, *IL-1*α (epithelial cell death signal), *ICAM1* (cell adhesion molecule), and *IRAK1* (signal transducing serine-threonine kinase in the IL1R and TLR pathways), were markedly upregulated in VEH/SIV rhesus macaques. The data confirms our recent demonstration of elevated *IRAK1* expression in the colons of chronically SIV-infected rhesus macaques consequent to reduced expression of its targeting miRNA (miR-150) during T cell activation (17). Consistent with high expression of IFN stimulated genes, it was not surprising to see upregulation of *SIKE1*, a negative regulator of TLR mediated IFN response gene expression. Interestingly, the expression of *CCL3 (MIP-1*α*)* and *CCL4-like* (MIP1-β) and macrophage secreted *CCL8 (MCP-2)*, all three with anti-HIV/SIV activity were significantly increased in the colons of THC/SIV rhesus macaques. Another anti-HIV chemokine, *CCL5*, was significantly downregulated in VEH/SIV but not THC/SIV rhesus macaques. Nevertheless, the most notable finding was the marked upregulation of anti-microbial defensin expression in colons of VEH/SIV but not THC/SIV rhesus macaques. Given that defensin expression is frequently increased in response to colonic inflammation and bacterial invasion of the barrier (43), the absence of anti-microbial defensin and miR-146b-5p/miR-130a upregulation suggests diminished intestinal inflammation and microbial translocation upon Δ^9^-THC treatment. Consistently, although statistically non-significant, THC/SIV rhesus macaques had lower plasma LBP levels compared to VEH/SIV rhesus macaques, suggesting decreased gut leak. These results concur with previous findings where long-term treatment with a CB2 receptor agonist reduced bacterial translocation in rats with cirrhosis and ascites (56).

Equally noteworthy is the finding that several genes regulating intestinal epithelial proliferation and homeostasis showed marked dysregulation in VEH/SIV but not in THC/SIV rhesus macaques. These included inhibitors of the Wnt/β-catenin signaling pathway (*WIF1, DKK1*, and *APC*), ATP-binding cassette (ABC) transporters *ABCB1* and *CFTR* and the central autophagy regulator *BECN1*. The ability of Δ^9^-THC to preserve luminal epithelial expression of *ABCB1* is noteworthy as this efflux pump was recently described to play a critical role in the constitutive luminal secretion of anti-inflammatory *N*-acylethanolamine type endocannabinoids that specifically suppressed transepithelial neutrophil migration and subsequent epithelial damage (57). As epithelial apoptosis is a hallmark of HIV/SIV infection (9), downregulation of negative regulators of the Wnt pathway may be a normal host response to enhance signaling to restore the barrier. Moreover, our group previously demonstrated reduced intestinal epithelial apoptosis (active caspase-3) in THC/SIV-infected rhesus macaques (58). Although T-regulatory (Treg) cells were not investigated in the present study, significantly high expression of *TNFSF18 (GITRL)*, a ligand expressed abundantly on the surface of intestinal Tregs (38) was detected in THC/SIV but not VEH/SIV rhesus macaques. Because the induction of Foxp3^+^ Tregs is another proposed mechanism by which cannabinoids exert their anti-inflammatory/immunosuppressive effects (8), the high expression of *TNFSF18* may indirectly reflect Treg mobilization by Δ^9^-THC, a mechanism that needs future validation. The potential for Treg mobilization in THC/SIV rhesus macaques is further supported by the high expression of *IL33*, which is produced by the intestinal epithelium and known to suppress inflammation by activating Tregs and type 2 innate lymphoid cell functions in the intestine (39, 40). Future experiments are needed to address this in detail.

The anti-inflammatory and epithelium protective properties of Δ^9^-THC became more conspicuous when we compared colonic gene expression between THC/SIV and VEH/SIV rhesus macaques. The critical differences that captured our attention were the complete absence of defensin upregulation (both *alpha* and *beta-defensins*) and decreased expression of *MMP*8 (12), *SOCS3*, and *C/EBP*γ (44, 45). Epithelium-specific genes that were upregulated in THC/SIV rhesus macaques included *KRT8* (46), an intermediate filament protein that regulates electrolyte transport, barrier function and offers efficient stress protection, *CLDN3* and *OCLN* (tight junction proteins), *PROM1* (marker of intestinal epithelial regeneration) (47), and anti-inflammatory/anti-apoptotic *MUC1*3 (48). Collectively, these novel findings provide evidence that cannabinoids preserve expression of genes linked to the maintenance of intestinal epithelial barrier integrity. Moreover, the finding that THC/SIV rhesus macaques had higher anti-HIV/SIV *CCL3, CCL4-like*, and *CCL5* mRNA expression is very intriguing as it raises the important question whether Δ^9^-THC preserves CD8^+^ T cell function by inhibiting excessive pro-inflammatory responses.

Consistent with *MMP8* downregulation in THC/SIV rhesus macaques, Nallathambi et al. (59) using HCT116 cells, showed that cannabinoids inhibited *TNF-*α induced upregulation of *MMP9*, another pro-inflammatory collagenase produced by intestinal epithelium and neutrophils (12). Computational analysis identified miR-204 (upregulated in THC/SIV rhesus macaques) to directly target *MMP8*. Using luciferase and overexpression assays, we confirmed miR-204 to target and downregulate *MMP8* expression, thus providing a molecular mechanism for its regulation. Surprisingly, treatment of Caco-2 cells with Δ^9^-THC did not increase miR-204 expression (Figure S6 in Supplementary Material), suggesting that Δ^9^-THC does not directly activate but may maintain physiological levels of miR-204 by inhibiting intestinal inflammation. This argument is compatible with the marked downregulation of miR-204 in *H. pylori*-induced duodenal ulcerative lesions (46). While we confirmed elevated miR-204 expression in CE, future studies employing cell sorting and *in situ* hybridization are needed to confirm the contributions of other cell types, especially, neutrophils to its high expression. These findings provide evidence that chronic cannabinoid treatment may prevent *MMP8* upregulation in the colon and one among several potential mechanisms includes preventing inflammation driven downregulation of miR-204. In addition, Δ^9^-THC treatment prevented chronic inflammation induced collagen deposition in lymph nodes suggesting that its anti-inflammatory effects are systemic and not restricted to the intestine. The absence of collagen deposition demonstrates the ability of Δ^9^-THC to preserve lymph node cortical morphology and architecture during SIV infection. The anti-fibrotic effects of cannabinoids have been shown to be mediated through activation of PPARγ (60), a molecular mechanism that we recently reported in the colon of chronically SIV-infected rhesus macaques (18). These findings also highlight the beneficial effects of complementing future HIV/SIV cure strategies with anti-inflammatory therapy to facilitate CD4^+^ T cell reconstitution in lymph nodes.

**Figure 12:**
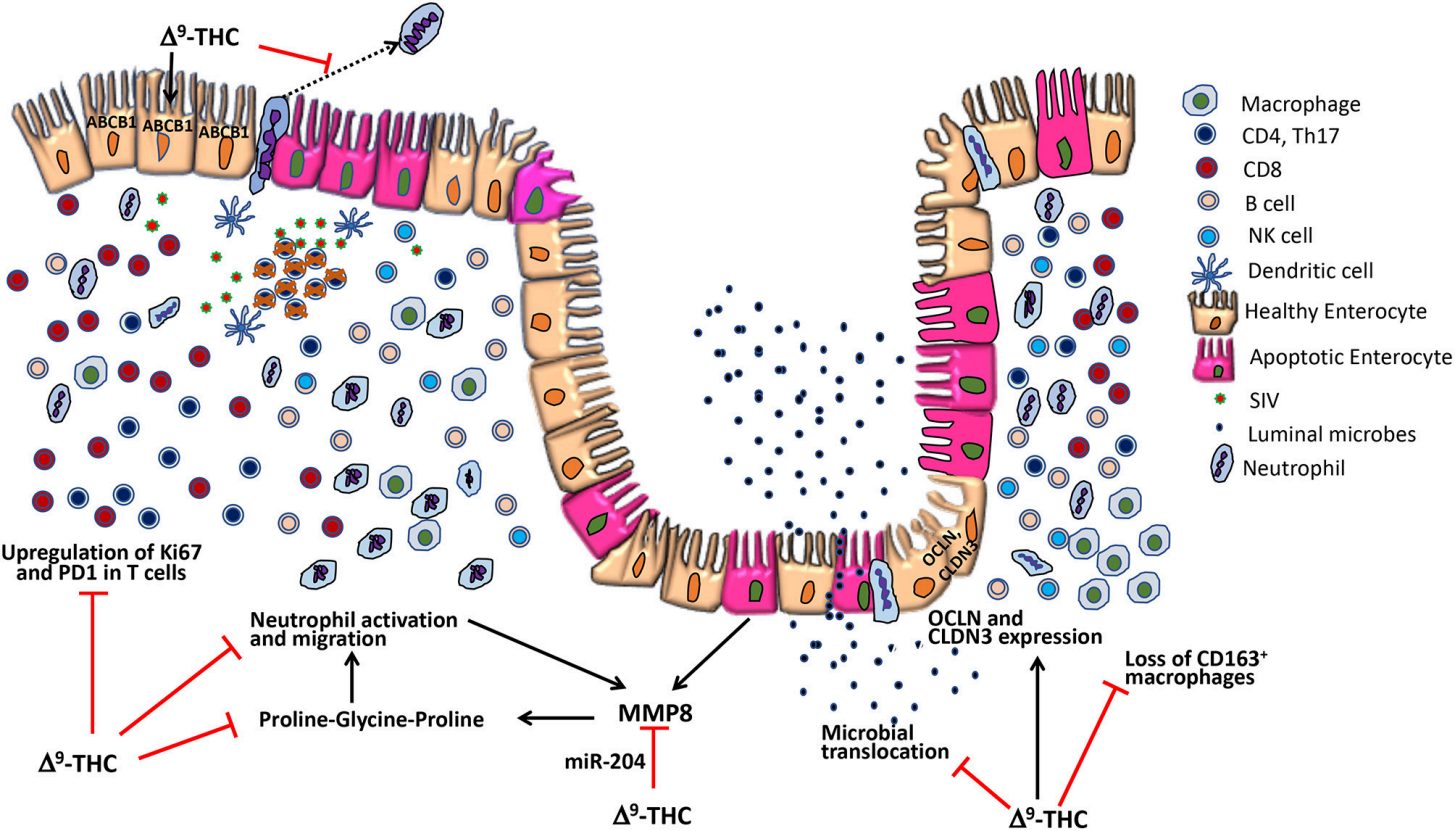
Potential anti-inflammatory mechanisms of delta-9-tetrahydrocannabinol (Δ^9^-THC) action in the intestine during HIV/SIV infection. During acute HIV/SIV infection, high viral replication in the intestine leads to massive CD4^+^ T-cell depletion resulting in pro-inflammatory cytokine production and subsequent T-cell hyperactivation. However, concurrent administration of cannabinoids (Δ^9^-THC) reduced CD4^+^ and CD8^+^ T-cell proliferation/activation (Ki67) and PD-1 expression. During intestinal inflammation, neutrophils migrate across the apical side of lining epithelium into the lumen, where they can cause damage to the epithelial cells (12). Δ^9^-THC can block neutrophil migration by acting via the CB2 receptor (9). Further, Δ^9^-THC prevented downregulation of ABCB1 (also called P-glycoprotein), an ATP-dependent drug efflux pump recently implicated in the luminal secretion of endocannabinoids that inhibited neutrophil activation and transmigration and subsequent epithelial damage (53) Furthermore, MMP-8 produced by epithelial cells and neutrophils degrades collagen to produce the tripeptide proline-glycine-proline, a strong neutrophil chemoattractant (12). Δ^9^-THC may prevent MMP-8 upregulation by epigenetic mechanisms by maintaining expression of miR-204, a miRNA that directly targets MMP8. Additionally, Δ^9^-THC increased the percentage of CD163^+^ anti-inflammatory macrophages and prevented downregulation of epithelial tight junction protein expression. Collectively, the anti-inflammatory effects of cannabinoids in the intestine may help maintain epithelial barrier integrity and prevent microbial translocation (8).

As the cannabinoid receptor, 2 (CB2) is expressed at high levels in immune cells and most evidence points toward its involvement in T cell recruitment and activation, we next investigated if Δ^9^-THC directly affected the expression of T cell activation markers, including PD-1 in the intestine during SIV infection. Although intestinal CD4^+^ T cell depletion was significant in both groups, the percentage of CD4^+^ T cells expressing the proliferation marker Ki67 within the surviving CD4^+^ T cell population was significantly reduced in THC/SIV rhesus macaques until 180 days post infection. These findings raise the important question of whether cannabinoids can inhibit the homeostatic proliferation of latently infected memory CD4^+^ T cell reservoirs under cART. Similarly, the percentage of CD8^+^/Ki67^+^ cells was significantly decreased in the intestines of THC/SIV rhesus macaques at 14 days post infection, suggesting that cannabinoids attenuate cytokine-driven activation induced CD8^+^ T cell proliferation during peak viral replication. Moreover, Δ^9^-THC effectively reduced the expression of PD-1, another important finding suggesting that cannabinoids may disrupt sustained PD-1 signaling implicated in intestinal CD4^+^ and CD8^+^ T cell exhaustion, a pathogenic event common to chronic viral infections (53). Unlike CD4^+^ T cells, the levels of PD-1 expression on CD8^+^ T cells are generally low in the intestine compared to peripheral blood and lymph nodes (61). This explains the low PD-1 expression on intestinal CD8^+^ T cells (Figure 8F). The zebra plot (Figures 8D,E) and the pseudocolor plot with smoothing (Figure S5) clearly showed that more cells stained positive with the PD-1 mAb than the control isotype control IgG. The PD-1 mAb clone EH12.EH7 (Biolegend) used in the current study has been used previously by other investigators to detect PD-1 expression on CD8^+^ T cells in lymph nodes of SIV-infected rhesus macaques (62) and in esophageal carcinomas in humans (63). These data demonstrate that the reactivity of the PD-1 mAb is specific. Therefore, we significantly detected differences in the levels of PD-1 in intestinal CD8^+^ T cells. Finally, THC/SIV rhesus macaques had a significantly higher percentage of anti-inflammatory CD163^+^ macrophages in their intestines at 180 days post-infection. These results suggest that cannabinoids can inhibit damaging inflammation and may help maintain gut homeostasis in HIV/SIV infection (8). Finally, total CD8^+^ T cell numbers were significantly reduced in peripheral blood of THC/SIV but not in VEH/SIV rhesus macaques demonstrating the ability of cannabinoids to prevent inflammation-induced CD8^+^ T cell expansion in peripheral blood. A schematic summarizing the anti-inflammatory effects of Δ^9^-THC in the intestine during the course of HIV/SIV infection is shown in Figure 12.

In summary, we demonstrate that long term, twice daily administration of Δ^9^-THC resulted in reduced expression of pro-inflammatory genes/micro-RNAs and inhibited T cell proliferation/activation without any adverse effects in cART-naive chronic SIV-infected rhesus macaques. More importantly, the anti-inflammatory effects extended to the lymph nodes, where extensive collagen deposition in B cell follicular regions was detected in VEH/SIV, but not in THC/SIV rhesus macaques. These novel findings together with recent legislation tolerating the medical use of marijuana suggest that future studies are warranted to determine whether supplementing cART with cannabinoids (non-psychotropic cannabidiol) can reduce residual intestinal inflammation and potentially the size of the gut-associated lymphoid tissue viral reservoir.

## Ethics Statement

Animal care, ethics, and experimental procedures. All experiments using rhesus macaques were approved by the Tulane and LSUHSC Institutional Animal Care and Use Committee (Protocol Nos- 3581 and 3781). The Tulane National Primate Research Center (TNPRC) is an Association for Assessment and Accreditation of Laboratory Animal Care International accredited facility (AAALAC #000594). The NIH Office of Laboratory Animal Welfare assurance number for the TNPRC is A3071-01. All clinical procedures, including administration of anesthesia and analgesics, were carried out under the direction of a laboratory animal veterinarian. Animals were anesthetized with ketamine hydrochloride for blood collection procedures. Intestinal pinch biopsies were collected by laboratory animal veterinarians. Animals were pre-anesthetized with ketamine hydrochloride, acepromazine, and glycopyrolate, intubated and maintained on a mixture of isoflurane and oxygen. All possible measures are taken to minimize discomfort of all the animals used in this study. Tulane University complies with NIH policy on animal welfare, the Animal Welfare Act, and all other applicable federal, state, and local laws.

## Author Contributions

The overall planning, direction, and design of the experiments were carried out by MM and PEM. VK, JM, and MM carried out the day-day sampling scheduling (animal experiments) and performed the microRNA profiling, RT-qPCR validation of miRNAs and genes of interest, immune precipitation/western blotting, and data analysis. BP designed the flow cytometry panels. WT and BP analyzed the flow cytometry data. CVS and BP performed the lipopolysaccharide binding protein ELISAs. SNB and MM performed the plasma and tissue viral load assays. JL performed the statistical analysis. PJD and XA did the Gomori one-step trichrome staining of lymph node sections, histopathological analysis, and quantification of collagen deposition. MM wrote the manuscript with input from all authors. PEM, PJD, WT, SNB, and BP provided helpful suggestions and review of the manuscript.

### Conflict of Interest Statement

The authors declare that the research was conducted in the absence of any commercial or financial relationships that could be construed as a potential conflict of interest.

## Supplementary Material

Figure S1 Normalized signal intensity **(A)** and delta-CT (ΔC_T_) **(B)** values for inflammation induced *MNP2* (also known as defensin alpha-2) in the colon of vehicle untreated, vehicle and THC treated SIV-infected and uninfected control rhesus macaques. The RT-qPCR assay was performed once. *MNP2* gene expression was normalized to β-Actin and relative changes in gene expression were calculated using the ΔΔC_T_ method. RT-qPCR data analysis using non-parametric Kruskal-Wallis test revealed differences among groups (*p* = 0.0001). *Post hoc* comparisons were performed using Dunn's test (a non-parametric test), with *p*-values adjusted using Benjamini-Hochberg method (*p* ≤ 0.05, FDR = 5%). The error bars represent standard error of mean fold change within each group. Due to the lack of availability of enough amounts of RNA, one animal (FT11) in the SIV-no vehicle group was not included in the RT-qPCR analysis shown in **(B)**. Note the high normalized signal intensity **(A)** and low delta-CT **(B)** values (increased mRNA expression) in vehicle treated and vehicle untreated SIV-infected rhesus macaques compared to THC/SIV and uninfected control rhesus macaques suggesting a lack of effect of vehicle on intestinal inflammatory gene expression.

Figure S2 Gating strategy of Ki67^+^ cells in duodenum lamina propria leukocytes from an SIV- infected rhesus macaque. Cells were gated first on singlets, CD45^+^ cells, followed by live cells and then on CD3^+^ T cells and subsequently on CD3/CD4^++^ and CD3/CD8^++^ T cell subsets. CD4^+^ T cells were further gated to quantify proliferating Ki67^+^ cells. The percentages of the total gated population are shown in each box of the plot. Note that the THC/SIV infected macaque had significantly fewer proliferating CD4^+^ T cells compared to the VEH/SIV infected macaque.

Figure S3 Plasma lipopolysaccharide binding protein (LBP) levels are relatively lower in THC-SIV **(B)** compared to VEH/SIV rhesus macaques. Plasma LBP levels were similar in both groups at 60 days post infection (days post infection) **(A)**. Within groups, the increase in plasma LBP concentration from 60 to 180 days post infection was greater in VEH/SIV (Avg 4.7–46 ng/mL) **(C)** compared to THC/SIV (Avg 4.4–21 ng/mL) **(D)** group. At both time points, plasma LBP concentrations remained undetectable (below 2 ng/ml) in 5/9 VEH/SIV and 2/7 THC/SIV rhesus macaques. Plasma samples were not available from A2L0694 (THC/SIV group). LBP data were analyzed using the Mann-Whitney *U*-test.

Figure S4 Gomori one-step trichrome stained lymph node sections of the remaining 3 out of 7 VEH/SIV **(A–C)** and 4 out of 7 THC/SIV rhesus macaques **(D–G)**. Note substantial blue staining collagen in the center of the lymphoid follicle in VEH/SIV **(A–C)** compared to THC/SIV rhesus macaques.

Figure S5 Gating strategy for PD-1 expression from a representative VEH/SIV rhesus macaque. Cells were gated first on singlets, lymphocytes, followed by live cells, and then on CD3^+^ T cells. CD3^+^ T-cells were further gated on CD3^+^CD4^+^ and CD3^+^CD8^+^ T cell subsets. The percentages of total PD-1 expression in both CD4^+^ and CD8^+^ T cells are shown in the bottom two rows where the positive PD-1 expression was compared to the isotype control (green histograms) both in the dot plots and histograms.

Figure S6 Δ^9^-THC does not upregulate miR-204 expression in Caco-2 cells. VEH- Vehicle, THC- Delta-9-tetrahydrocannabinol. miR-204 expression in Caco-2 cells was quantified using the individual TaqMan Stem-loop miRNA RT-qPCR assays. Treatments were performed in triplicate wells and repeated twice. miR-204 expression was normalized to RNU48 and difference between the two groups was analyzed using the Mann-Whitney *U*-test employing the Prism v5 software (GraphPad software). A *p*-value ≤0.05 was considered significant.

Table S1 CD4^+^ T cells are significantly depleted in the intestines of both VEH/SIV and THC/SIV rhesus macaques during chronic SIV infection. Table shows CD4^+^ and CD8^+^ T cell percentages in the intestine at necropsy. Normal CD4^+^ T cell percentages in the intestine as shown in Figure 7C range from 30 to 50%. The significantly low (1.7–19.2%) CD4^+^ T cell percentages in the intestine at necropsy suggests marked depletion of these cells. Also, note the concomitant increase in CD8^+^ T cell percentages.

Table S2 Raw CT, fold change (FC), and *P*-values of differentially expressed miRNAs in colon of VEH/SIV relative to control macaques.

Table S3 Raw CT, fold change (FC), and *P*-values of differentially expressed miRNAs in colon of THC/SIV relative to control macaques.

Table S4 Raw CT, fold change (FC), and *P*-values of differentially expressed miRNAs in colon of THC/SIV relative to VEH/SIV macaques.

Table S5 List of Upregulated genes in colon of VEH/SIV rhesus macaques compared to controls.

Table S6 List of Downregulated genes in colon of VEH/SIV rhesus macaques compared to controls.

Table S7 List of Upregulated genes in colon of THC/SIV rhesus macaques compared to controls.

Table S8 List of Downregulated genes in colon of THC/SIV rhesus macaques compared to controls.

Table S9 List of Downregulated genes in colon of THC/SIV compared to VEH/SIV rhesus macaques.

Table S10 List of Upregulated genes in colon of THC/SIV compared to VEH/SIV rhesus macaques.
