# Supplementary figures and images for: Cannabinoid Attenuation of Intestinal Inflammation in Chronic SIV-Infected Rhesus Macaques Involves T Cell Modulation and Differential Expression of Micro-RNAs and Pro-inflammatory Genes
Source: Front Immunol. 2019 Apr 30;10:914. doi: 10.3389/fimmu.2019.00914 (PMC6503054; doi:10.3389/fimmu.2019.00914)

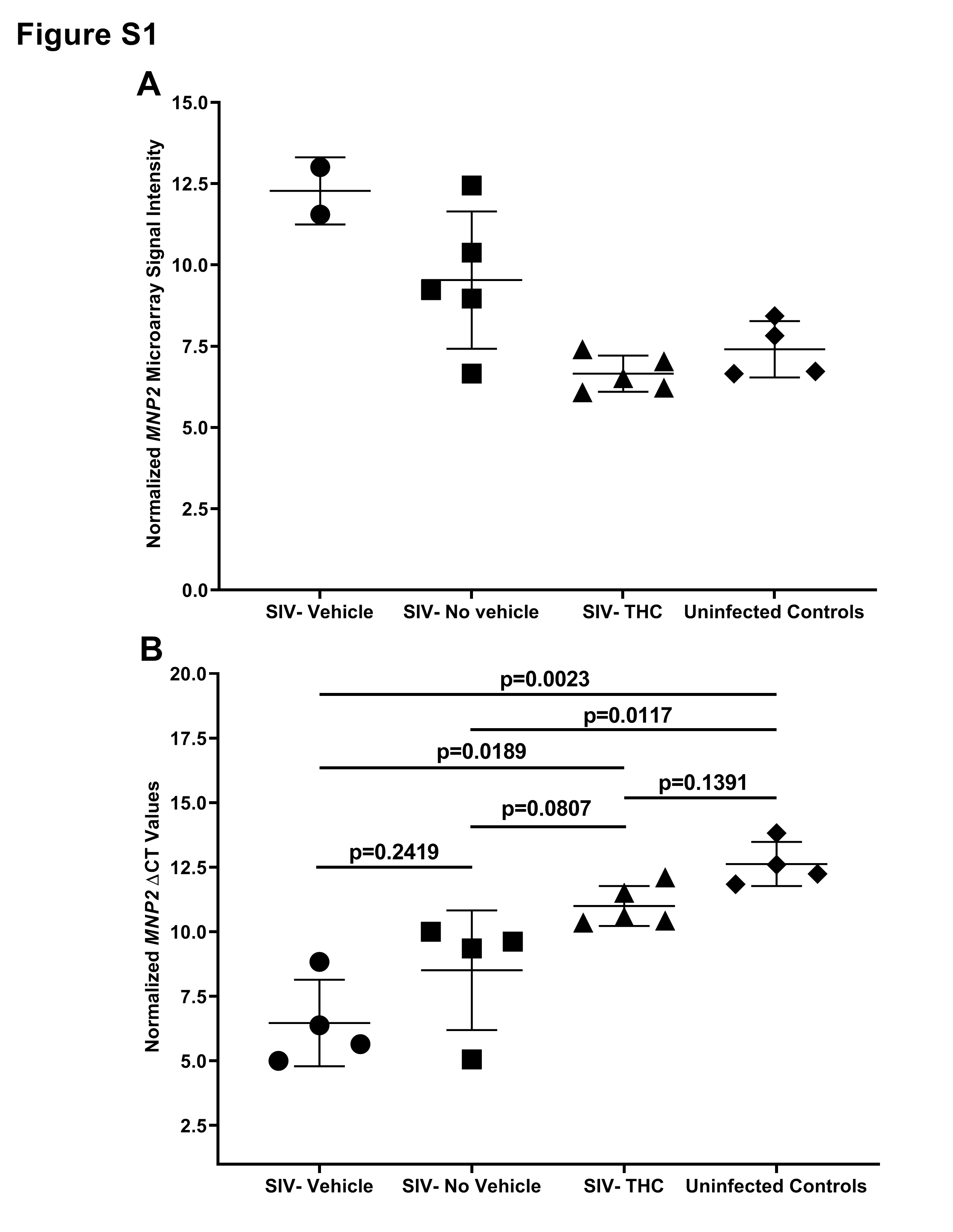

Supplement: Figure S1 — Normalized signal intensity (A) and delta-CT (ΔCT) (B) values for inflammation induced MNP2 (also known as defensin alpha-2) in the colon of vehicle untreated, vehicle and THC treated SIV-infected and uninfected control rhesus macaques. The RT-qPCR assay was performed once. MNP2 gene expression was normalized to β-Actin and relative changes in gene expression were calculated using the ΔΔCT method. RT-qPCR data analysis using non-parametric Kruskal-Wallis test revealed differences among groups (p = 0.0001). Post hoc comparisons were performed using Dunn's test (a non-parametric test), with p-values adjusted using Benjamini-Hochberg method (p ≤ 0.05, FDR = 5%). The error bars represent standard error of mean fold change within each group. Due to the lack of availability of enough amounts of RNA, one animal (FT11) in the SIV-no vehicle group was not included in the RT-qPCR analysis shown in (B). Note the high normalized signal intensity (A) and low delta-CT (B) values (increased mRNA expression) in vehicle treated and vehicle untreated SIV-infected rhesus macaques compared to THC/SIV and uninfected control rhesus macaques suggesting a lack of effect of vehicle on intestinal inflammatory gene expression. [file Image_1.TIF]

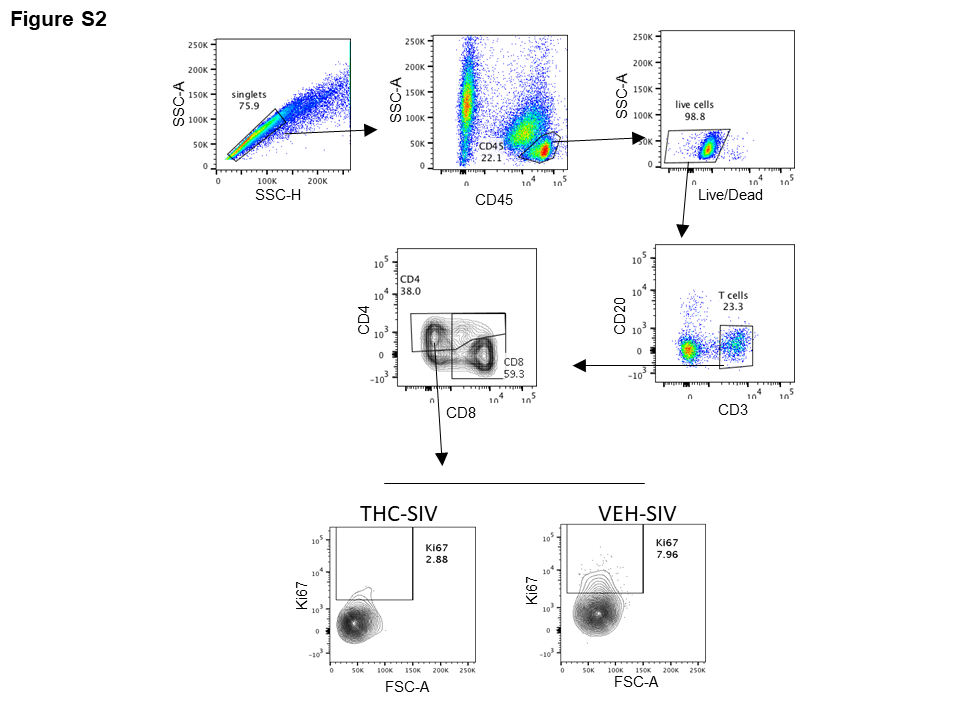

Supplement: Figure S2 — Gating strategy of Ki67+ cells in duodenum lamina propria leukocytes from an SIV- infected rhesus macaque. Cells were gated first on singlets, CD45+ cells, followed by live cells and then on CD3+ T cells and subsequently on CD3/CD4++ and CD3/CD8++ T cell subsets. CD4+ T cells were further gated to quantify proliferating Ki67+ cells. The percentages of the total gated population are shown in each box of the plot. Note that the THC/SIV infected macaque had significantly fewer proliferating CD4+ T cells compared to the VEH/SIV infected macaque. [file Image_2.TIF]

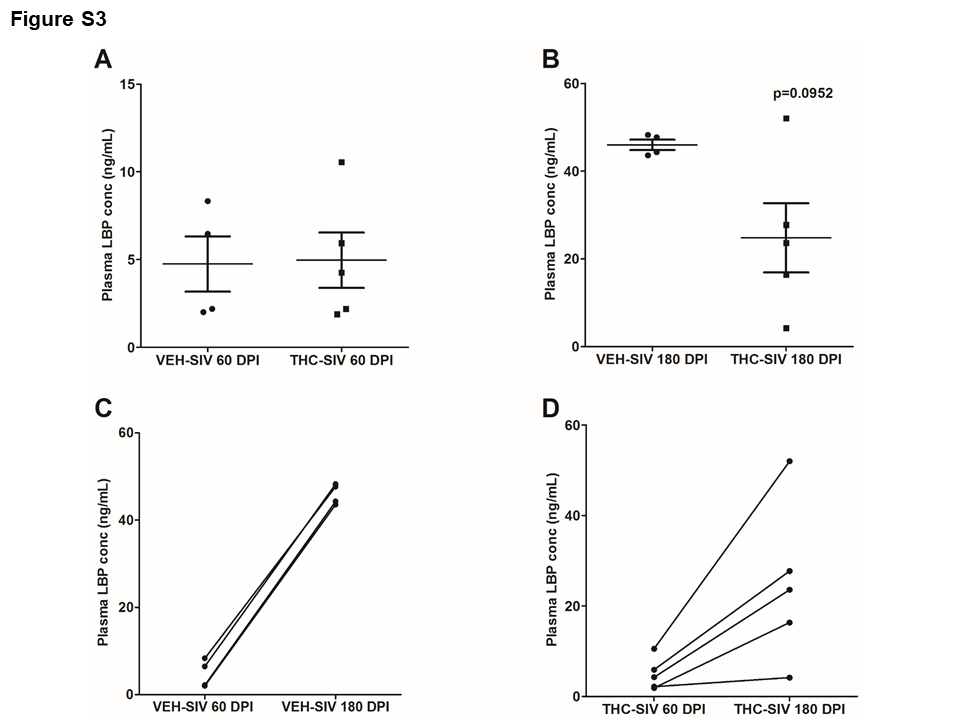

Supplement: Figure S3 — Plasma lipopolysaccharide binding protein (LBP) levels are relatively lower in THC-SIV (B) compared to VEH/SIV rhesus macaques. Plasma LBP levels were similar in both groups at 60 days post infection (days post infection) (A). Within groups, the increase in plasma LBP concentration from 60 to 180 days post infection was greater in VEH/SIV (Avg 4.7–46 ng/mL) (C) compared to THC/SIV (Avg 4.4–21 ng/mL) (D) group. At both time points, plasma LBP concentrations remained undetectable (below 2 ng/ml) in 5/9 VEH/SIV and 2/7 THC/SIV rhesus macaques. Plasma samples were not available from A2L0694 (THC/SIV group). LBP data were analyzed using the Mann-Whitney U-test. [file Image_3.TIF]

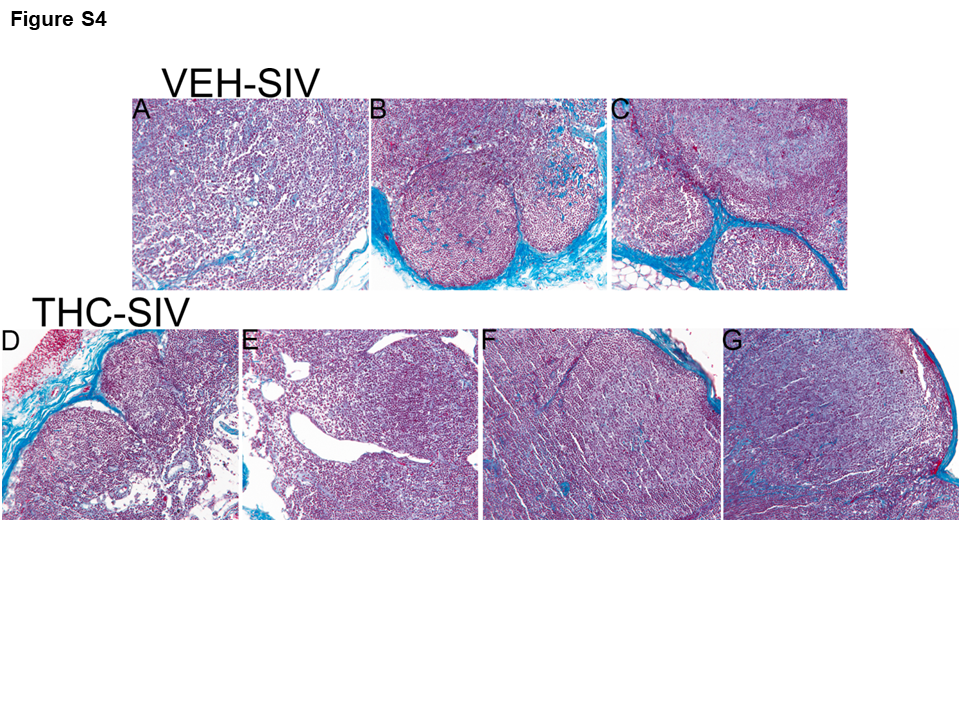

Supplement: Figure S4 — Gomori one-step trichrome stained lymph node sections of the remaining 3 out of 7 VEH/SIV (A–C) and 4 out of 7 THC/SIV rhesus macaques (D–G). Note substantial blue staining collagen in the center of the lymphoid follicle in VEH/SIV (A–C) compared to THC/SIV rhesus macaques. [file Image_4.TIF]

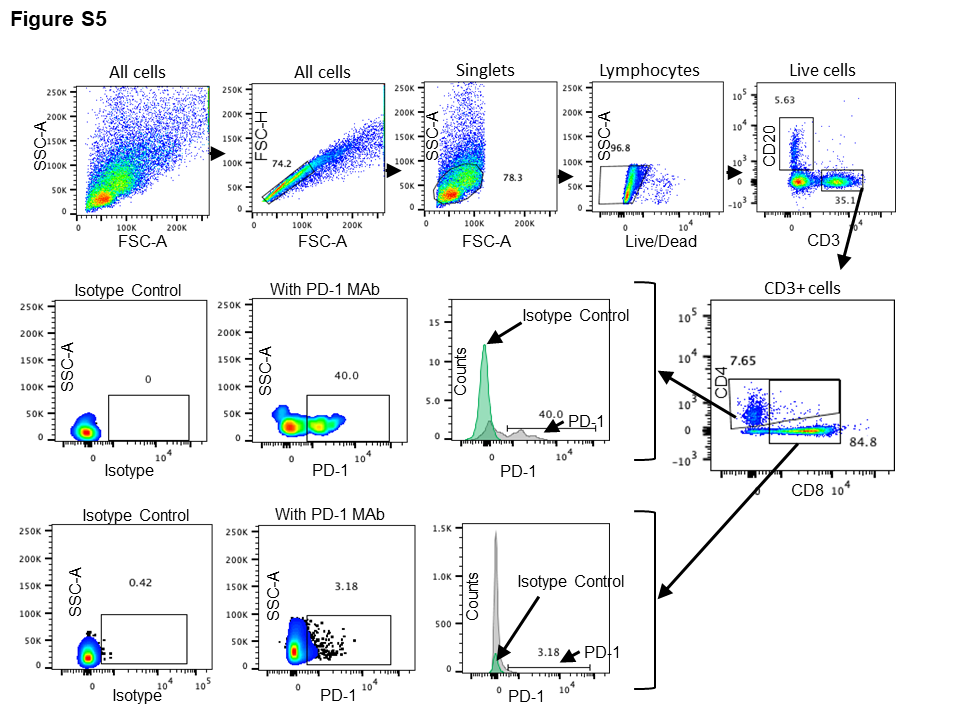

Supplement: Figure S5 — Gating strategy for PD-1 expression from a representative VEH/SIV rhesus macaque. Cells were gated first on singlets, lymphocytes, followed by live cells, and then on CD3+ T cells. CD3+ T-cells were further gated on CD3+CD4+ and CD3+CD8+ T cell subsets. The percentages of total PD-1 expression in both CD4+ and CD8+ T cells are shown in the bottom two rows where the positive PD-1 expression was compared to the isotype control (green histograms) both in the dot plots and histograms. [file Image_5.TIF]

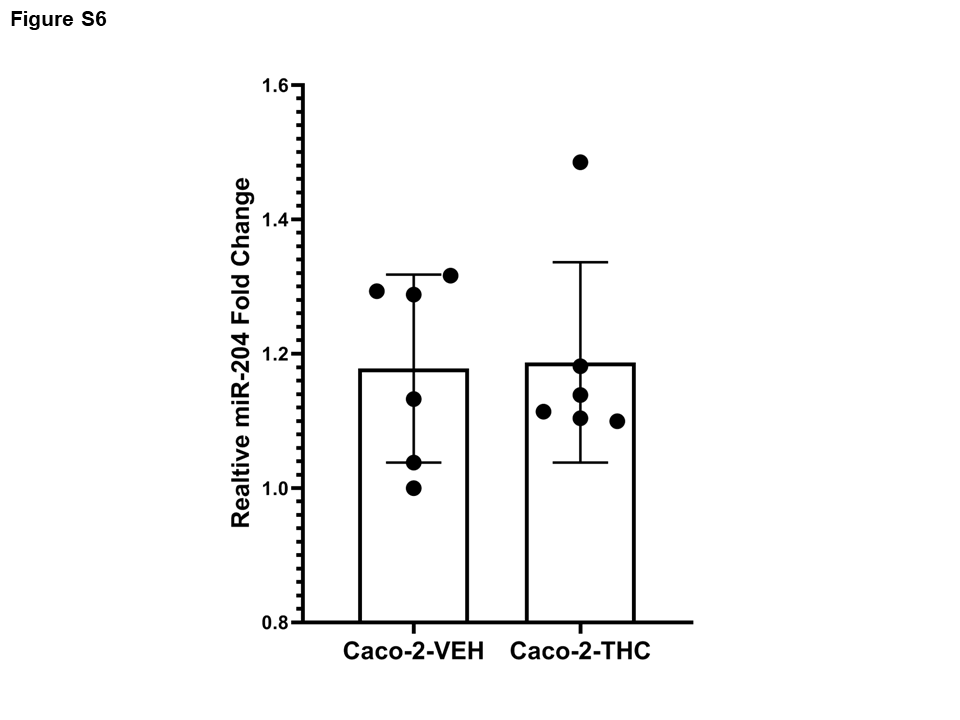

Supplement: Figure S6 — Δ9-THC does not upregulate miR-204 expression in Caco-2 cells. VEH- Vehicle, THC- Delta-9-tetrahydrocannabinol. miR-204 expression in Caco-2 cells was quantified using the individual TaqMan Stem-loop miRNA RT-qPCR assays. Treatments were performed in triplicate wells and repeated twice. miR-204 expression was normalized to RNU48 and difference between the two groups was analyzed using the Mann-Whitney U-test employing the Prism v5 software (GraphPad software). A p-value ≤0.05 was considered significant. [file Image_6.TIF]
