# Supplementary material for: Cannabinoid Attenuation of Intestinal Inflammation in Chronic SIV-Infected Rhesus Macaques Involves T Cell Modulation and Differential Expression of Micro-RNAs and Pro-inflammatory Genes
Source: Front Immunol. 2019 Apr 30;10:914. doi: 10.3389/fimmu.2019.00914 (PMC6503054; doi:10.3389/fimmu.2019.00914)
Supplement: Table S1 — CD4+ T cells are significantly depleted in the intestines of both VEH/SIV and THC/SIV rhesus macaques during chronic SIV infection. Table shows CD4+ and CD8+ T cell percentages in the intestine at necropsy. Normal CD4+ T cell percentages in the intestine as shown in Figure 7C range from 30 to 50%. The significantly low (1.7–19.2%) CD4+ T cell percentages in the intestine at necropsy suggests marked depletion of these cells. Also, note the concomitant increase in CD8+ T cell percentages. [file Data_Sheet_1.PDF]

Animal ID	$CD4^+$ T cell percentages	$CD8^+$ T cell percentages
**VEH/SIV**		
IH96	7.6	86.9
IN24	1.9	96.8
GA19	4.3	95.0
**THC/SIV**		
GV60	19.2	78.6
HT48	1.7	40.7
IA83	4.3	82.5
IH69	4.6	92.1
HI09	6.5	92.3
JB82	18.7	79.0

Table S1. $CD4^+$ T cells are significantly depleted in the intestines of both VEH/SIV and THC/SIV rhesus macaques during chronic SIV infection. Table shows $CD4^+$ and $CD8^+$ T cell percentages in the intestine at necropsy. Normal $CD4^+$ T cell percentages in the intestine as shown in figure 7C range from 30 to 50%. The significantly low (1.7 to 19.2%) $CD4^+$ T cell percentages in the intestine at necropsy suggests marked depletion of these cells. Also, note the concomitant increase in $CD8^+$ T cell percentages.
