# Supplementary material for: Cannabinoid Attenuation of Intestinal Inflammation in Chronic SIV-Infected Rhesus Macaques Involves T Cell Modulation and Differential Expression of Micro-RNAs and Pro-inflammatory Genes
Source: Front Immunol. 2019 Apr 30;10:914. doi: 10.3389/fimmu.2019.00914 (PMC6503054; doi:10.3389/fimmu.2019.00914)
Supplement: Table S2 — Raw CT, fold change (FC), and P-values of differentially expressed miRNAs in colon of VEH/SIV relative to control macaques. [file Data_Sheet_2.PDF]

Table S2. Raw CT, fold change (FC) and P values of differentially expressed miRNAs in colon of VEH/SIV relative to control macaques

	Uninfected Controls (n=6)						VEH/SIV (n=9)										
miRNA ID	EL66	EH70	EH80	HT22	HF54	HR42	IH96	HV48	IN24	JC81	FT11	GH25	HB31	GA19	HD08-90D	FC	P.Value
miR-656	24.7	24.6	23.9	22.7	22.7	24.3	24.0	24.5	40.0	24.4	26.0	25.7	26.7	25.3	25.2	-6.4	0.0048
miR-654	24.8	25.5	24.1	26.6	23.8	25.0	25.2	25.1	25.6	25.2	26.7	26.1	26.9	40.0	28.5	-5.1	0.0256
miR-661	21.8	22.4	20.1	21.8	17.3	19.4	22.3	23.8	22.3	22.6	23.2	22.4	21.4	23.4	22.9	-3.6	0.0256
miR-376c	19.3	19.6	18.6	18.7	17.8	18.2	20.0	19.7	20.8	19.0	20.5	20.9	21.2	20.2	21.3	-2.5	0.0008
miR-708	21.6	21.4	20.5	19.9	20.0	20.7	21.3	22.0	23.4	20.9	22.8	22.7	22.7	22.2	22.3	-2.3	0.0016
miR-337-3p	25.9	26.7	26.2	25.7	25.7	26.3	26.1	26.7	27.6	27.2	29.3	27.5	27.8	29.1	27.5	-2.2	0.0028
miR-885-5p	24.6	25.3	24.2	25.0	22.9	24.4	24.8	23.8	28.3	24.7	27.6	25.6	26.2	25.4	26.8	-2.2	0.0256
miR-199b	22.5	22.5	22.2	21.4	20.8	21.8	22.7	22.1	23.6	23.2	24.7	23.8	23.7	23.9	22.2	-2.1	0.0016
miR-369-3p	26.5	27.4	25.8	24.9	25.7	26.0	27.8	27.3	28.3	27.1	28.4	26.1	26.9	27.0	27.9	-2.0	0.0360
miR-410	22.4	22.7	21.8	21.3	21.0	21.8	22.1	22.5	23.7	22.2	23.8	23.7	23.8	23.4	23.3	-1.9	0.0016
miR-744*	26.0	26.1	25.7	25.7	24.7	26.1	25.7	26.0	28.1	25.2	28.6	27.9	27.9	27.1	26.4	-1.8	0.0496
miR-422a	24.0	24.7	23.8	23.7	22.6	23.6	24.7	23.6	26.4	23.8	25.7	25.5	25.4	24.6	24.6	-1.7	0.0028
miR-137	23.8	23.6	22.9	22.9	23.6	24.6	23.9	23.1	25.8	25.4	25.7	25.3	24.9	24.7	23.8	-1.7	0.0360
miR-328	20.5	20.6	20.4	19.4	19.0	19.5	20.5	18.8	22.8	20.3	22.5	21.0	21.8	21.2	20.6	-1.7	0.0496
miR-487b	23.8	24.1	23.3	22.7	22.1	23.2	23.1	23.5	24.8	23.8	25.6	24.9	25.2	24.4	24.0	-1.7	0.0028
miR-107	23.7	23.9	22.6	22.5	21.9	22.8	23.8	23.6	24.1	23.2	25.8	24.0	24.6	23.6	23.7	-1.7	0.0256
miR-149	20.9	20.8	20.4	19.6	19.7	20.4	20.5	19.9	22.2	20.8	22.4	21.9	21.4	21.2	22.3	-1.7	0.0120
miR-889	24.1	24.5	23.6	22.9	22.7	24.4	24.0	24.6	25.4	23.8	25.8	25.2	25.1	24.6	24.1	-1.6	0.0120
miR-27b	18.2	18.0	17.8	16.8	16.5	17.8	17.7	17.1	19.3	18.1	19.7	19.0	19.2	18.5	18.4	-1.6	0.0048
miR-340	21.8	22.0	21.2	21.4	21.2	21.9	21.9	21.4	23.4	21.8	23.8	22.9	23.7	22.7	21.8	-1.6	0.0076
miR-98	20.4	20.1	20.5	19.2	18.9	19.5	20.8	19.5	21.4	20.9	21.8	21.2	20.7	20.2	20.6	-1.5	0.0496
miR-221	19.1	19.8	18.9	18.4	17.9	18.7	19.5	18.2	20.8	18.8	21.4	19.7	20.0	19.8	19.8	-1.5	0.0360
miR-145	12.1	11.8	11.5	11.0	10.8	11.9	11.8	10.4	13.8	11.9	13.9	12.9	12.8	12.3	12.6	-1.5	0.0256
miR-411	19.6	20.3	18.9	18.8	18.6	19.5	19.3	19.6	20.5	19.6	20.9	20.6	20.7	20.5	20.2	-1.5	0.0048
miR-181a	18.5	18.7	17.8	18.0	16.8	17.8	18.3	17.7	19.2	18.1	19.8	18.8	19.6	18.9	18.8	-1.4	0.0120
miR-365	19.6	20.0	19.3	19.3	18.8	19.7	19.6	18.0	21.8	20.7	21.6	20.4	20.8	19.8	19.8	-1.4	0.0120
miR-148a	18.4	19.1	18.0	17.8	17.5	18.0	19.1	17.9	19.2	18.5	18.9	19.3	19.6	19.3	18.9	-1.4	0.0176
miR-139-5p	18.4	18.8	17.8	17.5	17.0	18.3	18.5	16.7	20.2	18.5	20.2	18.9	19.1	18.4	18.7	-1.4	0.0360
miR-301	21.5	22.1	21.0	20.5	20.7	21.8	21.8	21.7	22.1	21.7	21.8	22.5	23.2	21.7	21.8	-1.3	0.0496
miR-99a	17.2	17.3	16.4	16.1	15.3	16.7	16.8	15.8	18.4	16.8	18.4	17.7	17.7	17.1	16.8	-1.3	0.0496
miR-193b*	24.9	25.6	25.5	24.6	24.4	25.3	25.7	24.7	23.2	25.8	27.3	26.4	26.7	26.1	26.2	-1.3	0.0256
miR-30b	14.6	14.8	14.4	13.8	13.3	14.4	14.6	13.6	15.7	14.5	15.8	14.9	15.6	14.7	14.5	-1.2	0.0496
miR-454	20.0	20.8	19.6	19.7	19.2	20.9	19.8	18.7	20.6	19.8	21.0	20.2	20.7	20.0	20.2	1.2	0.0360
miR-152	19.9	20.4	19.7	19.8	19.1	20.9	19.8	18.7	20.7	19.7	20.6	20.2	20.6	19.9	19.7	1.3	0.0360
miR-455	22.0	22.5	21.7	21.2	21.1	21.9	21.8	20.8	22.5	21.2	21.9	22.3	22.6	21.7	21.3	1.3	0.0256
miR-18a	21.6	22.7	21.5	21.3	21.4	21.9	21.4	21.4	21.9	21.7	22.3	21.8	22.1	21.3	21.5	1.3	0.0256
miR-1201	23.8	24.9	23.7	23.8	23.2	24.4	23.8	22.8	24.3	23.1	24.8	24.2	24.8	23.9	23.5	1.4	0.0048
miR-185	23.5	23.8	22.7	22.7	22.5	23.8	22.8	22.6	23.4	22.1	23.3	23.6	23.8	23.1	22.8	1.4	0.0076
miR-361-3p	23.9	25.2	23.5	23.1	22.7	24.1	23.5	22.8	23.9	22.8	24.7	23.8	24.4	23.3	23.3	1.4	0.0120
miR-130a	18.8	19.0	18.5	18.6	18.7	20.1	18.4	17.5	19.6	18.7	19.5	18.8	19.7	18.6	18.3	1.5	0.0496
miR-452	23.2	23.9	23.0	23.7	22.8	24.3	22.8	22.3	23.9	23.0	23.8	23.3	24.0	23.0	22.7	1.6	0.0028
miR-362	21.2	21.6	20.8	22.1	21.7	22.6	21.0	20.1	21.8	20.8	22.7	21.3	21.7	21.4	21.8	1.6	0.0360
miR-382	20.3	21.7	19.9	21.0	19.9	21.5	19.9	19.9	21.2	19.9	21.0	20.6	20.7	20.1	20.3	1.6	0.0076
miR-432	27.2	27.3	25.5	26.1	25.0	26.5	25.5	25.8	25.7	24.7	26.8	26.1	26.7	26.3	25.7	1.7	0.0256
miR-25	19.9	20.7	19.6	20.7	20.5	21.8	20.0	19.2	20.7	19.9	20.8	20.3	20.5	20.0	20.2	1.7	0.0496
miR-942	20.7	21.5	20.6	20.8	20.4	22.0	20.2	20.3	20.8	20.1	21.3	21.3	20.9	19.9	21.1	1.7	0.0176
miR-425-5p	19.9	20.2	20.2	19.8	18.9	20.1	19.3	19.3	19.6	19.0	20.3	19.6	20.3	18.9	19.0	1.7	0.0048
miR-18b	22.9	22.9	22.8	21.6	21.8	22.3	21.1	21.7	22.1	21.8	22.6	22.2	22.7	21.6	22.0	1.7	0.0048
miR-29b	20.9	22.1	21.2	21.8	21.3	23.1	21.8	20.0	21.3	21.6	21.2	21.8	22.3	20.8	21.0	1.8	0.0496
miR-146b	17.5	17.8	16.4	16.1	15.5	16.6	16.1	16.3	16.7	15.8	15.8	16.4	16.8	15.8	15.8	1.8	0.0048
miR-502-3p	24.1	24.4	23.5	24.6	23.7	25.8	23.8	22.7	24.3	23.8	24.8	23.7	24.2	23.8	23.4	1.8	0.0028
miR-222	16.8	17.5	16.7	15.6	15.2	15.9	16.1	14.7	16.3	15.9	15.8	15.7	16.6	15.4	15.1	1.9	0.0120
miR-19b	13.6	14.3	13.3	14.8	14.4	15.8	13.4	13.0	14.1	13.8	14.2	13.8	14.7	13.7	13.6	1.9	0.0496
miR-200b*	20.5	21.8	20.5	20.7	19.6	21.6	20.2	19.0	19.1	19.8	21.9	20.3	20.6	20.0	20.2	2.1	0.0028
miR-29b-2*	27.0	27.4	30.1	25.6	26.8	27.1	25.5	25.5	26.6	26.0	27.2	27.8	27.6	26.6	26.6	2.1	0.0496
miR-331	17.7	18.4	17.7	19.2	18.8	20.9	18.0	16.8	19.1	17.8	18.7	17.9	18.6	17.5	17.6	2.3	0.0360
miR-500	22.9	23.4	22.5	23.0	23.7	24.7	22.6	21.3	22.9	22.7	23.6	22.3	22.8	22.3	22.3	2.4	0.0008
miR-106a	14.8	15.6	14.6	16.6	15.8	17.5	14.7	14.2	15.2	14.6	15.7	15.0	15.4	14.7	15.0	2.4	0.0076
miR-200a	16.5	17.0	16.6	20.0	16.8	18.6	16.8	15.6	16.1	16.8	17.3	16.7	17.5	16.5	16.8	2.5	0.0496
miR-190b	24.7	25.7	25.1	25.4	23.8	26.3	23.5	23.8	23.4	23.4	22.7	23.5	23.8	22.8	22.0	5.2	0.0004
miR-592	26.4	29.3	27.7	25.7	28.5	31.3	26.1	25.3	26.9	26.1	25.7	25.8	26.3	26.8	25.5	5.6	0.0120
