# Supplementary material for: Cannabinoid Attenuation of Intestinal Inflammation in Chronic SIV-Infected Rhesus Macaques Involves T Cell Modulation and Differential Expression of Micro-RNAs and Pro-inflammatory Genes
Source: Front Immunol. 2019 Apr 30;10:914. doi: 10.3389/fimmu.2019.00914 (PMC6503054; doi:10.3389/fimmu.2019.00914)
Supplement: Table S3 — Raw CT, fold change (FC), and P-values of differentially expressed miRNAs in colon of THC/SIV relative to control macaques. [file Data_Sheet_3.PDF]

Table S3. Raw CT, fold change (FC) and P values of differentially expressed miRNAs in colon of THC/SIV relative to control macaques

	Uninfected Controls (n=6)						THC/SIV (n=8)									
miRNA ID	EL66	EH70	EH80	HT22	HF54	HR42	A2L069	GV60	HT48	IA83	IH69	IA04	HI09	JB82	FC	P.Value
miR-657	17.9	17.6	18.8	15.8	17.5	15.3	18.8	40.0	17.8	40.0	40.0	18.4	20.4	40.0	-4.5	0.0047
miR-181c	23.8	24.2	23.6	21.6	20.8	22.4	22.7	24.2	24.4	23.9	25.8	23.1	24.5	23.8	-2.3	0.0293
miR-378	24.7	25.5	23.4	23.5	22.8	24.3	24.1	26.8	25.1	25.8	25.2	24.7	24.7	24.7	-1.9	0.0200
miR-199b	22.5	22.5	22.2	21.4	20.8	21.8	21.5	23.7	24.4	23.8	23.8	20.8	22.7	21.7	-1.7	0.0293
miR-221	19.1	19.8	18.9	18.4	17.9	18.7	18.6	20.2	20.5	20.4	20.0	18.6	19.4	19.4	-1.7	0.0027
miR-410	22.4	22.7	21.8	21.3	21.0	21.8	21.7	22.8	23.4	23.6	23.5	21.5	22.8	22.2	-1.7	0.0007
miR-720	14.8	14.0	13.7	13.4	13.8	14.2	14.4	15.5	15.1	15.3	15.7	13.1	14.8	14.8	-1.6	0.0127
miR-708	21.6	21.4	20.5	19.9	20.0	20.7	20.4	22.0	22.3	22.8	21.8	20.5	21.8	20.7	-1.6	0.0127
miR-598	24.7	24.9	24.9	24.1	23.5	25.0	23.8	25.9	24.9	25.8	26.5	23.7	26.7	25.4	-1.6	0.0426
miR-194	15.1	15.6	14.8	15.2	13.7	14.6	15.2	15.2	16.0	15.8	16.4	16.4	15.3	14.8	-1.6	0.0426
miR-107	23.7	23.9	22.6	22.5	21.9	22.8	22.7	23.3	24.6	23.8	24.8	23.0	23.6	23.5	-1.5	0.0293
miR-214	18.7	18.6	18.3	17.8	17.6	18.2	18.5	19.2	18.3	19.8	19.7	17.6	19.6	18.8	-1.5	0.0200
miR-889	24.1	24.5	23.6	22.9	22.7	24.4	23.8	24.7	25.4	25.5	25.0	22.9	24.5	23.6	-1.5	0.0200
miR-106b	17.2	18.1	17.3	16.5	15.9	16.7	17.1	17.7	17.7	18.0	18.4	17.4	17.8	17.3	-1.5	0.0426
miR-148a	18.4	19.1	18.0	17.8	17.5	18.0	18.2	19.7	18.9	19.5	19.2	17.8	18.8	18.5	-1.5	0.0047
miR-337-5p	24.9	24.8	24.5	24.0	23.5	24.4	24.1	25.3	25.2	26.1	25.6	23.2	25.8	24.7	-1.4	0.0293
miR-340	21.8	22.0	21.2	21.4	21.2	21.9	20.5	23.0	22.9	22.6	23.5	21.1	22.6	21.8	-1.4	0.0293
miR-301	21.5	22.1	21.0	20.5	20.7	21.8	20.5	22.1	22.5	22.5	23.1	20.6	22.2	21.4	-1.4	0.0127
miR-495	22.0	22.0	21.5	21.6	20.6	21.3	21.1	22.1	22.7	22.8	23.4	20.6	22.4	21.7	-1.4	0.0426
miR-491	22.5	22.8	22.8	21.8	21.0	22.2	21.7	22.9	23.4	23.7	23.7	21.8	22.6	22.2	-1.3	0.0293
miR-411	19.6	20.3	18.9	18.8	18.6	19.5	19.5	20.7	19.9	20.7	20.8	18.5	19.8	19.0	-1.3	0.0200
let-7g	17.8	18.2	17.5	17.5	16.4	17.5	16.8	18.4	18.1	18.7	18.8	16.9	18.0	17.7	-1.2	0.0200
miR-191	14.6	15.3	14.3	14.7	13.9	15.3	13.8	14.7	14.8	15.2	15.5	13.8	14.7	14.1	1.2	0.0200
miR-454	20.0	20.8	19.6	19.7	19.2	20.9	18.8	20.2	20.1	21.0	19.4	18.8	19.8	19.4	1.4	0.0127
miR-185	23.5	23.8	22.7	22.7	22.5	23.8	22.1	23.2	23.2	23.3	23.2	21.6	22.8	22.5	1.5	0.0007
miR-200b*	20.5	21.8	20.5	20.7	19.6	21.6	19.2	20.6	20.4	20.2	20.7	20.7	20.5	19.5	1.6	0.0200
miR-382	20.3	21.7	19.9	21.0	19.9	21.5	20.0	20.5	20.6	20.6	20.3	18.8	20.7	19.6	1.7	0.0200
miR-425-5p	19.9	20.2	20.2	19.8	18.9	20.1	18.8	19.8	19.2	19.8	19.9	18.7	19.1	19.0	1.7	0.0007
miR-1300	27.7	27.3	27.3	27.8	27.6	27.7	26.8	27.7	27.0	26.9	27.1	24.5	27.1	26.7	2.0	0.0080
miR-500	22.9	23.4	22.5	23.0	23.7	24.7	21.8	22.5	22.5	22.7	23.6	21.5	22.6	22.3	2.1	0.0007
miR-27a	18.7	19.0	18.4	19.7	18.8	21.5	17.5	18.8	19.3	19.2	19.7	16.9	18.4	17.6	2.1	0.0293
miR-106a	14.8	15.6	14.6	16.6	15.8	17.5	14.4	14.8	14.9	15.3	15.6	14.8	14.8	14.4	2.1	0.0293
