# Supplementary material for: Cannabinoid Attenuation of Intestinal Inflammation in Chronic SIV-Infected Rhesus Macaques Involves T Cell Modulation and Differential Expression of Micro-RNAs and Pro-inflammatory Genes
Source: Front Immunol. 2019 Apr 30;10:914. doi: 10.3389/fimmu.2019.00914 (PMC6503054; doi:10.3389/fimmu.2019.00914)
Supplement: Table S4 — Raw CT, fold change (FC), and P-values of differentially expressed miRNAs in colon of THC/SIV relative to VEH/SIV macaques. [file Data_Sheet_4.PDF]

Table S4. Raw CT, fold change (FC) and P values of differentially expressed miRNAs in colon of THC/SIV relative to VEH/SIV macaques

	VEH/SIV (n=9)									THC/SIV (n=8)									
miRNA ID	IH96	HV48	IN24	JC81	FT11	GH25	HB31	GA19	HD08-90D	A2L0694	GV60	HT48	IA83	IH69	IA04	HI09	JB82	FC	P.Value
miR-592	26.1	25.3	26.9	26.1	25.7	25.8	26.3	26.8	25.5	26.6	29.9	26.7	27.8	28.5	25.0	27.1	26.2	-2.7	0.0037
miR-96	25.8	25.2	25.9	26.7	28.2	25.3	26.3	25.8	25.5	25.8	25.9	28.8	27.5	28.3	26.8	26.4	26.1	-2.2	0.0206
miR-21	14.8	13.9	14.9	14.6	14.4	14.2	15.0	13.9	13.8	14.7	15.5	14.1	15.3	15.8	14.4	15.0	14.5	-1.7	0.0079
miR-141	18.6	17.2	19.0	18.7	18.4	17.7	19.6	18.5	18.1	18.2	18.8	19.2	19.1	19.5	18.7	18.8	18.4	-1.6	0.0274
miR-222	16.1	14.7	16.3	15.9	15.8	15.7	16.6	15.4	15.1	15.8	16.4	15.4	16.9	17.4	14.7	16.5	15.7	-1.5	0.0274
miR-455	21.8	20.8	22.5	21.2	21.9	22.3	22.6	21.7	21.3	21.5	22.2	22.5	23.2	23.6	20.6	22.0	21.4	-1.5	0.0079
miR-151-5P	22.7	21.7	23.8	22.5	24.2	23.1	24.4	22.8	22.6	22.6	23.8	23.1	23.4	24.8	22.3	23.6	22.9	-1.4	0.0274
miR-19b-1*	25.6	25.2	25.5	25.7	26.8	26.1	26.3	26.0	25.6	25.5	26.1	26.4	26.7	26.0	25.0	26.9	25.8	-1.3	0.0152
miR-324-5p	21.4	20.6	22.0	21.0	23.1	21.9	22.6	21.3	21.6	20.4	21.5	22.1	21.8	22.1	19.8	21.2	20.8	1.2	0.0206
miR-328	20.5	18.8	22.8	20.3	22.5	21.0	21.8	21.2	20.6	19.4	21.0	20.6	21.0	21.7	18.5	20.5	20.1	1.4	0.0360
miR-204	18.8	18.1	19.9	18.5	20.4	20.5	20.4	18.8	19.5	17.6	18.7	18.7	19.8	20.8	17.2	18.8	17.6	1.5	0.0274
miR-422a	24.7	23.6	26.4	23.8	25.7	25.5	25.4	24.6	24.6	22.6	24.0	24.7	24.8	25.2	23.8	24.3	23.6	1.5	0.0152
miR-744*	25.7	26.0	28.1	25.2	28.6	27.9	27.9	27.1	26.4	25.0	25.9	27.0	26.8	26.1	25.6	25.4	26.0	1.7	0.0274
miR-1290	22.8	22.8	23.8	23.2	26.6	24.1	23.8	24.9	22.7	21.8	24.5	24.7	22.8	22.9	21.2	22.6	21.8	1.8	0.0360
miR-1300	26.4	28.5	27.8	27.4	28.0	29.1	28.7	27.5	28.1	26.8	27.7	27.0	26.9	27.1	24.5	27.1	26.7	2.0	0.0274
miR-885-5p	24.8	23.8	28.3	24.7	27.6	25.6	26.2	25.4	26.8	22.8	24.5	24.6	25.8	27.2	21.6	25.4	24.4	2.2	0.0464
miR-656	24.0	24.5	40.0	24.4	26.0	25.7	26.7	25.3	25.2	23.7	24.8	25.6	25.7	25.0	23.1	24.5	23.8	4.3	0.0274
miR-135b*	21.1	21.0	21.3	21.2	40.0	22.8	22.2	21.8	21.6	20.9	21.6	20.8	20.8	21.2	19.5	20.8	20.6	6.3	0.0079
