# Supplementary material for: Cannabinoid Attenuation of Intestinal Inflammation in Chronic SIV-Infected Rhesus Macaques Involves T Cell Modulation and Differential Expression of Micro-RNAs and Pro-inflammatory Genes
Source: Front Immunol. 2019 Apr 30;10:914. doi: 10.3389/fimmu.2019.00914 (PMC6503054; doi:10.3389/fimmu.2019.00914)
Supplement: Table S5 — List of Upregulated genes in colon of VEH/SIV rhesus macaques compared to controls. [file Data_Sheet_5.PDF]

Table S5. List of Upregulated genes in colon of VEH/SIV rhesus macaques compared to controls

Gene Symbol	Gene Name	Fold Change	P value
Inflammatory signaling			
S100A8	S100 calcium binding protein A8	30.7	0.0084
IL8	Interleukin 8	6.5	0.0309
CCL2	Chemokine (C-C motif) ligand 2	1.8	0.0437
ICAM1	Intercellular adhesion molecule 1	3.3	0.0268
IL1A	Interleukin 1, alpha	5.7	0.0456
IRAK1	Interleukin-1 receptor-associated kinase 1	3.3	0.0093
IL21R	interleukin 21 receptor	1.8	0.0444
Intestinal Anti-Microbial Defense			
LOC574310	alpha-defensin 3 precursor	13.5	0.0362
DEFA4	defensin, alpha 4, corticostatin	10.4	0.0424
ROAD2	oral alpha defensin 2	8.9	0.0257
LOC574383	alpha-defensin 6 precursor	7.5	0.0358
MNP2	alpha-defensin 2	5.5	0.0263
DEFB2L	b-defensin2-like	3.1	0.0215
DEFB108B	defensin, beta 108B	9.8	0.0004
Regulation of Interferon Signaling			
IFI6	interferon, alpha-inducible protein 6	8.2	0.0002
IFI27	ISG12(c) protein-like	8.1	0.0011
IFIT1	interferon-induced protein with tetratricopeptide repeats 1	7.9	0.0005
MX1	myxovirus (influenza virus) resistance 1	6.5	0.0078
MX2	myxovirus (influenza virus) resistance 2	4.3	0.0005
IFIT2	Interferon-induced protein with tetratricopeptide repeats 2	4.1	0.0059
ISG15	ISG15 ubiquitin-like modifier	2.4	0.0231
IFIT5	interferon-induced protein with tetratricopeptide repeats 5	1.6	0.0261
IRF7	interferon regulatory factor 7	2.1	0.0022
IRF9	interferon regulatory factor 9	2.1	0.0031
STAT1	signal transducer and activator of transcription 1, 91kDa	1.5	0.0176
SIKE1	suppressor of IKBKE 1	30.7	0.0084
Anti-HIV Signaling			
CCL3	chemokine (C-C motif) ligand 3	2.9	0.0288
SDF-1/CXCL12	Stromal derived factor-1	1.8	0.0443
