# Supplementary material for: Cannabinoid Attenuation of Intestinal Inflammation in Chronic SIV-Infected Rhesus Macaques Involves T Cell Modulation and Differential Expression of Micro-RNAs and Pro-inflammatory Genes
Source: Front Immunol. 2019 Apr 30;10:914. doi: 10.3389/fimmu.2019.00914 (PMC6503054; doi:10.3389/fimmu.2019.00914)
Supplement: Table S6 — List of Downregulated genes in colon of VEH/SIV rhesus macaques compared to controls. [file Data_Sheet_6.PDF]

Table S6. List of Downregulated genes in colon of VEH/SIV rhesus macaques compared to controls

Gene Symbol	Gene Name	Fold Change	P value
Anti-HIV Signaling			
CCL5	chemokine (C-C motif) ligand 5	2.7	0.0218
Immune/Inflammatory response			
Mamu-DRB	Mamu-DRB mRNA for major histocompatibility complex class II	12.8	0.0001
LOC714202	complement component (3d/Epstein Barr virus) receptor 2	60.1	0.0151
HDAC6	histone deacetylase 6	1.5	0.0489
IGF1R	insulin-like growth factor 1 receptor	1.6	0.0460
CXCR6	chemokine (C-X-C motif) receptor 6	3.9	0.0301
ECM remodeling			
CAPN13	calpain 13	43.9	0.0031
SERPINA9	serpin peptidase inhibitor, clade A member 9	14.4	0.0024
Epithelial proliferation and DIfferentiation			
STK31	serine/threonine kinase 31	13.1	0.0190
WIF1	WNT inhibitory factor 1	12.1	0.0001
DKK1	dickkopf 1 homolog	5.1	0.0436
APC	adenomatous polyposis coli	1.9	0.0479
RORC	RAR-related orphan receptor C	2.1	0.0122
WT1	Wilms tumor 1	2.0	0.0474
DTX4	deltex homolog 4	2.4	0.0337
FZD1	Frizzled-1	1.8	0.0037
Ion Transport			
ABCB1	ATP-binding cassette, sub-family B (MDR/TAP), member 1	4.8	0.0313
CFTR	cystic fibrosis transmembrane conductance regulator	1.9	0.0065
Anti-microbial/Anti-Inflammatory Signaling			
CCL21	chemokine (C-C motif) ligand 21	7.2	0.0088
IRF5	Interferon regulatory factor 5	1.9	0.0092
NLRP3	NLR family, pyrin domain containing 3	1.5	0.0436
FASLG	Fas ligand (TNF superfamily, member 6)	1.6	0.0496
BECN1	Beclin-1	1.4	0.0389
