# Supplementary material for: Cannabinoid Attenuation of Intestinal Inflammation in Chronic SIV-Infected Rhesus Macaques Involves T Cell Modulation and Differential Expression of Micro-RNAs and Pro-inflammatory Genes
Source: Front Immunol. 2019 Apr 30;10:914. doi: 10.3389/fimmu.2019.00914 (PMC6503054; doi:10.3389/fimmu.2019.00914)
Supplement: Table S7 — List of Upregulated genes in colon of THC/SIV rhesus macaques compared to controls. [file Data_Sheet_7.PDF]

Table S7. List of Upregulated genes in colon of THC/SIV rhesus macaques compared to controls

Gene Symbol	Gene Name	Fold Change	P value
Inflammatory signaling			
S100A8	S100 calcium binding protein A8	21.1	0.0289
IL8	interleukin 8	11.1	0.0047
IL34	interleukin 34	2.1	0.0417
Anti-Inflammatory signaling			
IL33	interleukin 33	1.9	0.0478
TNFSF18	tumor necrosis factor (ligand) superfamily, member 18	5.7	0.0115
Anti-Microbial Signaling			
CXCL10	chemokine (C-X-C motif) ligand 10	16.6	0.0294
CXCL14	chemokine (C-X-C motif) ligand 14	1.4	0.0250
Anti-HIV/SIV Signaling			
CCL3	chemokine (C-C motif) ligand 3	4.7	0.0075
CCL8	chemokine (C-C motif) ligand 8	4.7	0.0228
CCL4L1	chemokine (C-C motif) ligand 4-like 1	1.9	0.0270
Interferon Signaling			
IFIT1B	interferon-induced protein with tetratricopeptide repeats 1B	11.3	0.0407
MX1	myxovirus (influenza virus) resistance 1	6.3	0.0033
IFI27	ISG12(c) protein-like	5.9	0.0079
IFIT1	interferon-induced protein with tetratricopeptide repeats 1	5.6	0.0332
STAT1	signal transducer and activator of transcription 1, 91kDa	4.0	0.0456
IRF2	interferon regulatory factor 2	1.7	0.0379
IRF9	interferon regulatory factor 9	2.6	0.0447
Autophagy			
DRAM1	DNA-damage regulated autophagy modulator 1	2.4	0.0457
ATGR12	autophagy related 12	1.5	0.0157
