# Supplementary material for: Cannabinoid Attenuation of Intestinal Inflammation in Chronic SIV-Infected Rhesus Macaques Involves T Cell Modulation and Differential Expression of Micro-RNAs and Pro-inflammatory Genes
Source: Front Immunol. 2019 Apr 30;10:914. doi: 10.3389/fimmu.2019.00914 (PMC6503054; doi:10.3389/fimmu.2019.00914)
Supplement: Table S8 — List of Downregulated genes in colon of THC/SIV rhesus macaques compared to controls. [file Data_Sheet_8.PDF]

Table S8. List of Downregulated genes in colon of THC/SIV rhesus macaques compared to controls

Gene Symbol	Gene Name	Fold Change	P value
ECM Remodeling			
CAPN13	calpain 13	16.5	0.0457
ELANE	elastase, neutrophil expressed	1.8	0.0245
Immune/Inflammatory response			
IL20RB	interleukin 20 receptor beta	2.3	0.0398
IL2	interleukin 2	2.2	0.0085
IRF8	interferon regulatory factor 8	1.4	0.0364
GATA2	GATA binding protein 2	2.2	0.0120
CTLA4	cytotoxic T-lymphocyte-associated protein 4	2.1	0.0203
Anti-microbial/Anti-Inflammatory Signaling			
NLRP11	NLR family, pyrin domain containing 11	5.4	0.0124
Defensin Processing			
PRSS2	protease, serine, 2	4.0	0.0282
Apoptosis			
PRKCB	Protein kinase C	1.9	0.0218
XAF1	XIAP associated factor 1	1.9	0.0357
IL24	interleukin-24-like	2.1	0.0152
CIDEC	cell death-inducing DFFA-like effector c	2.1	0.0464
EBF3	early B-cell factor 3	1.7	0.0209
Anti-HIV Signaling			
CXCL12	chemokine (C-X-C motif) ligand 12	1.3	0.0223
