# Supplementary material for: Cannabinoid Attenuation of Intestinal Inflammation in Chronic SIV-Infected Rhesus Macaques Involves T Cell Modulation and Differential Expression of Micro-RNAs and Pro-inflammatory Genes
Source: Front Immunol. 2019 Apr 30;10:914. doi: 10.3389/fimmu.2019.00914 (PMC6503054; doi:10.3389/fimmu.2019.00914)
Supplement: Table S9 — List of Downregulated genes in colon of THC/SIV compared to VEH/SIV rhesus macaques. [file Data_Sheet_9.PDF]

Table S9. List of Downregulated genes in colon of THC/SIV compared to VEH/SIV rhesus macaques

Gene Symbol	Gene Name	Fold Change	P value
Intestinal Anti-Microbial Defense			
ROAD-1	oral alpha defensin 1	21.3	0.0106
ROAD-2	oral alpha defensin 2	21.6	0.0040
LOC574309	alpha-defensin 2 precursor	19.1	0.0163
MNP2	alpha-defensin 2	22.6	0.0105
LOC574310	alpha-defensin 3 precursor	14.9	0.0094
DEFA4	defensin, alpha 4, corticostatin	24.2	0.0262
LOC574382	alpha-defensin 5 precursor	17.6	0.0134
LOC574383	alpha-defensin 6 precursor	15.9	0.0031
DEFB2L	b-defensin2-like	12.2	0.0261
DEFB108B	defensin, beta 108B	3.5	0.0360
Intestinal Defensin Processing			
PRSS2	protease, serine, 2 (trypsin 2)	2.5	0.0407
Anti-Inflammatory signaling			
GSTT2	glutathione S-transferase theta 2	2.6	0.0270
GSTA4	Glutathione S-transferase	3.0	0.0226
HIF3A	hypoxia inducible factor 3, alpha subunit	2.5	0.0434
SIKE1	suppressor of IKBKE 1	1.4	0.0362
Nutrient/Drug Transporter			
ABCC11	ATP-binding cassette, sub-family C (CFTR/MRP)	2.5	0.0473
ABCB9	ATP-binding cassette, sub-family B (MDR/TAP), member 9	4.2	0.0383
FABP6	fatty acid binding protein 6, ileal	3.1	0.0052
FKBP5	FK506 binding protein 5	2.4	0.0117
Inflammatory Signaling			
MMP8	matrix metallopeptidase 8 (neutrophil collagenase)	2.4	0.0327
NCF1	neutrophil cytosolic factor 1	2.2	0.0344
CSF3R	colony stimulating factor 3 receptor (granulocyte)	2.2	0.0037
CARD9	caspase recruitment domain family, member 9	1.8	0.0034
SOCS3	Suppressor of cytokine signaling 3	3.0	0.0515
CEBPG	CCAAT/enhancer binding protein (C/EBP), gamma	1.3	0.0334
SHH	sonic hedgehog	2.6	0.0385
Apoptosis			
TNFSF15	tumor necrosis factor (ligand) superfamily, member 15	2.4	0.0240
DEDD	death effector domain containing	1.9	0.0309
