# Supplementary material for: Cannabinoid Attenuation of Intestinal Inflammation in Chronic SIV-Infected Rhesus Macaques Involves T Cell Modulation and Differential Expression of Micro-RNAs and Pro-inflammatory Genes
Source: Front Immunol. 2019 Apr 30;10:914. doi: 10.3389/fimmu.2019.00914 (PMC6503054; doi:10.3389/fimmu.2019.00914)
Supplement: Table S10 — List of Upregulated genes in colon of THC/SIV compared to VEH/SIV rhesus macaques. [file Data_Sheet_10.PDF]

Table S10. List of Upregulated genes in colon of THC/SIV compared to VEH/SIV rhesus macaques

Gene Symbol	Gene Name	Fold Change	P value
Epithelial proliferation and DIfferentiation			
CLCA4	chloride channel accessory 4	15.4	0.0412
PROM1	prominin 1	9.0	0.0499
WIF1	WNT inhibitory factor 1	7.2	0.0113
KRT8	keratin 8	5.3	0.0347
KRT7	keratin 7	2.0	0.0214
CFTR	cystic fibrosis transmembrane conductance regulator	3.5	0.0436
ABCB1	ATP-binding cassette, sub-family B (MDR/TAP), member 1	4.5	0.0546
Anti-microbial/Anti-Inflammatory Signaling			
NLRP7	NLR family, pyrin domain containing 7	3.4	0.0315
NLRP3	NLR family, pyrin domain containing 3	1.8	0.0032
TLR4	toll-like receptor 4	3.0	0.0366
PRDX3	peroxiredoxin 3	1.9	0.0368
TNFSF18	tumor necrosis factor (ligand) superfamily, member 18	4.2	0.0109
Anti-HIV Signaling			
CCL5	isolate 95112 chemokine ligand 5	2.2	0.0122
CXCL12	chemokine (C-X-C motif) ligand 12	3.6	0.0453
CCR1	chemokine (C-C motif) receptor 1	2.0	0.0470
GZMB	granzyme B	1.8	0.0481
Epithelial Barrier Integrity			
MUC13	mucin 13, cell surface associated	6.5	0.0428
CLDN3	Claudin 3	1.4	0.0290
OCLN	Occludin	1.3	0.0398
LOC716409	coxsackie virus and adenovirus receptor	2.7	0.0338
CLDN23	claudin 23	5.7	0.0258
